# Improved Broad Spectrum Antifungal Drug Synergies with Cryptomycin, a Cdc50-Inspired Antifungal Peptide

**DOI:** 10.1021/acsinfecdis.4c00681

**Published:** 2024-10-29

**Authors:** Robert J. Tancer, Siddhi Pawar, Yina Wang, Cristina R. Ventura, Gregory Wiedman, Chaoyang Xue

**Affiliations:** † Public Health Research Institute and Department of Microbiology, Biochemistry, and Molecular Genetics, New Jersey Medical School, Rutgers University, Newark, New Jersey 07103, United States; ‡ Department of Chemistry and Biochemistry, Seton Hall University, South Orange, New Jersey 07079, United States

**Keywords:** antifungal peptide, drug resistance, pathogenic fungi, cdc50, lipid flippase

## Abstract

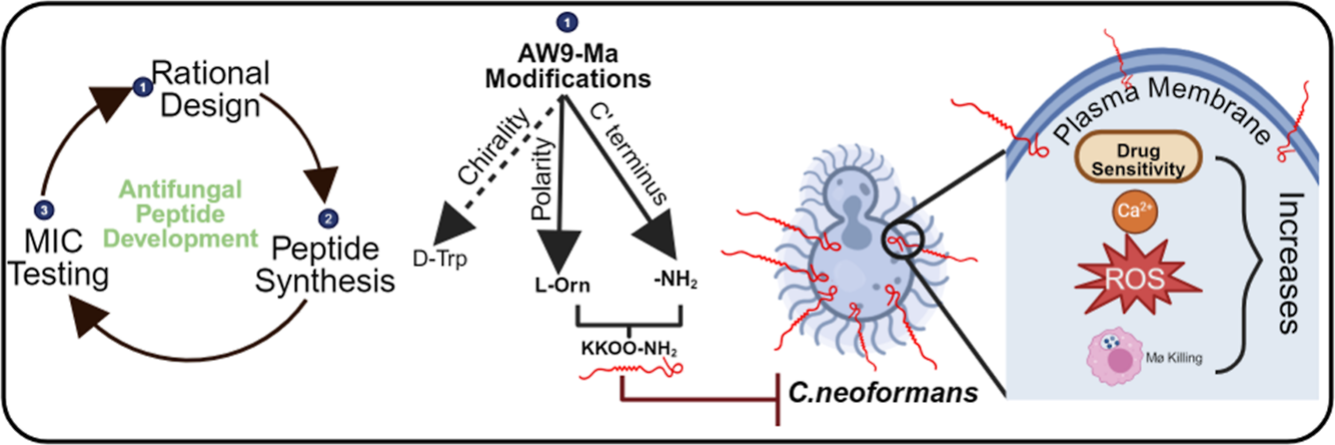

Fungal infections in humans are difficult to treat, with very limited drug options. Due to a confluence of factors, there is an urgent need for innovation in the antifungal drug space, particularly to combat increasing antifungal drug resistance. Our previous studies showed that Cdc50, a subunit of fungal lipid translocase (flippase), is essential for *Cryptococcus neoformans* virulence and required for antifungal drug resistance, suggesting that fungal lipid flippase could be a novel drug target. Here, we characterized an antifungal peptide, Cryptomycinamide (KKOO-NH_2_), derived from a 9-amino acid segment of the *C. neoformans* Cdc50 protein. A fungal killing assay indicated that KKOO-NH_2_ is fungicidal against *C. neoformans*. The peptide has antifungal activity against multiple major fungal pathogens with a minimum inhibitory concentration (MIC) of 8 μg/mL against *C. neoformans* and *Candida glabrata*, 16 μg/mL against *Candida albicans* and *C. auris*, and 32 μg/mL against *Aspergillus fumigatus*. The peptide has low cytotoxicity against host cells based on our hemolysis assays and vesicle leakage assays. Strikingly, the peptide exhibits strong drug synergy with multiple antifungal drugs, including amphotericin B, itraconazole, and caspofungin, depending on the specific species on which the combinations were assayed. The fluorescently labeled peptide was detected to localize to the plasma membrane, likely inhibiting key interactions of Cdc50 with membrane proteins such as P4 ATPases. *Cryptococcus* cells exposed to sub-MIC of peptide showed increased reactive oxygen species production and intracellular calcium levels, indicating a peptide-induced stress response. Decreased intracellular proliferation within macrophages was observed after 30 min of peptide exposure and 24 h coincubation with macrophages, providing a potential translational mechanism to explore further in vivo. In aggregate, the synergistic activity of our KKOO-NH_2_ peptide may offer a potential novel candidate for combination therapy with existing antifungal drugs.

## Challenge of Medically Relevant Fungal Pathogens

A multitude of economic, social, and environmental factors have coalesced, leading to ongoing increases in antifungal drug resistance in fungal pathogens. Economically, many pharmaceutical companies have abandoned antimicrobial development all together due to high risk and low potential return on investment.^1^ Socially—there are increasing numbers of susceptible individuals in the population due to age and increasing rates of comorbidities. Environmentally—climate change and agricultural use of fungicides to reduce food spoilage lead to selective pressure toward evolving more virulent and drug-resistant strains, respectively.^2^

Fungi are eukaryotic organisms that share conserved cellular mechanisms with humans and animals. This evolutionary proximity in the tree of life means there are fewer biochemical differences to exploit for safe and effective antifungal activity.^3^ The three main classes of antifungals, azoles, echinocandins, and polyenes, all have benefits and challenges associated with their use.^4^ Azoles like fluconazole (FLC) and itraconazole (ITR) exert their fungistatic effects by inhibiting ergosterol biosynthesis but require long-term administration, which can lead to resistance.^5^ Echinocandins like caspofungin (CAS) inhibit the biosynthesis of the key cell wall component β-1,3-glucan. They are well tolerated by the human host; however, resistance can occur, and some species like *Cryptococcus neoformans* (*Cn*), are inherently resistant to echinocandins.^6^ Polyenes make up the most powerful antifungal class. Polyenes bind ergosterol in the plasma membrane (PM) and form pores, causing cytoplasm leakage and cell death.^7^ However, clinical use of polyenes like amphotericin B (AMB) is impeded by harsh side effects and challenging drug–drug interactions with other medications.^8,9^

Active areas of research and development involve creating new classes of antifungal compounds that exert effects in monotherapy.^10^ Other research approaches endeavor to develop targets associated with drug resistance and virulence for combination or adjuvant therapy. Additional strategies include delivery vehicle formulation aimed at increasing stability and bioavailability while reducing toxicity of existing compounds.^11,12^ Orthogonal approaches include fungal vaccination strategies to immunize the host against fungal pathogenesis.^13−16^

### Flippases Contribute to Antifungal Drug Resistance and Pathogenicity

Membrane proteins, particularly phospholipid flippases, are an emerging drug target of interest in pathogens like *C. neoformans*, *Candida* spp., and *Aspergillus* spp. because studies have linked both α and β subunits of these proteins with in vitro drug resistance, virulence factors, and pathogenicity in animal models. Therefore, flippases provide a promising conserved drug target that could help to sensitize fungal cells to existing therapeutics and reduce pathogenicity in vivo.^17−27^

Lipid flippase is the common name for P4 ATPase, a type of phospholipid translocator that translocate lipids from the exocytoplasmic side of the membrane to the cytofacial side of the membrane. Lipid flippases are conserved in eukaryotes and are composed of 10 transmembrane (TM) helices in the catalytic α subunit and two TM helices in the noncatalytic β subunit.^28^ P4 ATPases are classified as either P4A or P4B.^29^ P4A ATPases require the β-subunit protein, Cdc50 and its homologues, and P4B ATPases are composed only of the catalytic α-subunit. In the absence of the β-subunit, P4A ATPases do not leave the endoplasmic reticulum (ER) and do not form functional complexes with ATP.^30−34^

Our previous studies have identified that the *Cn cdc50*Δ mutant was highly susceptible to CAS compared to the wild-type.^23^ The minimum inhibitory concentration (MIC) of CAS was reduced 4-fold from 16 to 4 μg/mL when Cdc50 was deleted. The surprising observation that loss of Cdc50 sensitized *Cryptococcus* to CAS led to an obvious question: why did a mutation to a seemingly disparate cellular system-membrane homeostasis proteins have such a dramatic effect on the susceptibility to cell wall biosynthesis inhibitors? It was subsequently discovered that Cdc50 in *C. neoformans* is unique because it has also been shown to form a functional complex with “Caspofungin resistant mutant 1” (Crm1), a mechanosensitive calcium ion channel. Crm1’s location in the ER membrane regulates CAS resistance in *C. neoformans* by modulating intracellular calcium levels.^35,36^ It was hypothesized that in combination with an ATPase, “Cdc50 may coordinate Ca^2+^ efflux” from the PM and participate in calcium homeostasis during stress response.^35^ Studies have also shown *cdc50*Δ is sensitized to other antifungals, like azoles and macrophage killing in vitro.^23^ The precise mechanism of Cdc50-mediated antifungal drug resistance is unclear; however, it may be related to changes in either lipid distribution in the PM, by changes in stress response due to altered calcium homeostasis, or possibly both.^23,35^ Similar to *Cn cdc50*Δ, both *Candida albicans* (*Ca*) and *Candida glabrata* (*Cg*) *cdc50*Δ knockouts have also demonstrated pronounced CAS sensitivity making Cdc50 an ideal drug target to address antifungal drug resistance.^18,19^

### Leveraging Knowledge of cdc50Δ Susceptibility for Lead Antifungal Peptide Development

We endeavored to develop an inhibitor to target flippase function in *Cn* and harness the critical importance of Cdc50 in antifungal drug resistance and virulence. The initial effort resulted in the antifungal peptide (AFP), “Myristic acid WGGIKKNYA” (AW9-Ma), which was derived from a segment of the *Cn* Cdc50 protein that possessed some antifungal activity against *C. neoformans* H99 with a MIC of 64 μg/mL, additive drug synergy with CAS, and a fractional inhibition concentration (FIC) index of 0.5. The peptide sequence was identified using an epitope approach. First, anti-Cdc50 polyclonal antibodies were generated. The antibody originating from a segment called sequence 41 of Cdc50 was capable of localizing to the PM of wild-type yeast cells in spheroplast form, while the antibody generated from a segment called sequence 1 of Cdc50 was not capable of binding to the PM. Interestingly, when synthesized as N′ myristylated peptides and tested for antifungal activity, peptides based on Cdc50 sequence 1 possessed some antifungal activity, while peptides based on sequence 41 possessed no antifungal activity. The 9-amino acid backbone “WGGIKKNYA” was identified from a truncation scan of the original sequence 1 peptide. A fatty acid tail (FAT) scan of the 9-amino acid peptide backbone identified in the truncation scan confirmed myristic acid was the optimal FAT length.^37^

Several key questions remained regarding the published peptide: what additional sequence modifications can further improve the antifungal activity of the peptide? Whether the peptide sensitizes fungi to other antifungal compounds, and which ones? And finally, what is the potential effective range against other fungal pathogens in addition to *Cn*, either by the peptide itself or in combination with other antifungals?^37,38^

In our previous study, we began probing the structure–activity relationship (SAR) of the peptide and found that substituting a lysine residue, a source of a cationic charge, with alanine, a nonpolar amino acid, led to a dramatic reduction in the peptide activity. In this study, we assessed if substituting lysine residues with a more polar source of a cationic charge would impart superior activity; lysine residues were substituted with l-ornithine (O), l-diaminobutyric acid (Dab), and l-diaminopropionic acid (Dap). It has been reported that substituting lysine residues with ornithine residues in peptides enhanced their stability and activity.^39−41^ Furthermore, peptides containing ornithine residues should exhibit decreased protease degradation compared to the peptide’s unmodified counterpart due to its non-proteogenic nature, providing additional justification to employ such modifications here.^39−41^ Next, we endeavored to assess whether epimerization of any single chiral α amino group would alter activity. Finally, the effect of carboxy terminus modifications, including amide (–NH_2_) and methylamide (–NHMe) substitution on peptide activity was assessed. In aggregate, this study identified a much-improved peptide-based inhibitor with a broad antifungal activity spectrum in combination with either triazoles or AMB, which provides a foundation for further development of this peptide.

## Results and Discussion

### Cryptomycinamide Inhibits the Growth of Multiple Key Fungal Pathogens In Vitro

First, the MICs of the peptides and commonly used antifungal drugs were assessed on their own. Key peptides and standard antifungal MICs are reported in Table 1. A full accounting of MICs of intermediate SAR screen peptides is reported in Supporting Information, Table S1. Most single compound MIC data was unremarkable and in-line with reported values.^42^*C. auris*, *Aspergillus fumigatus* (*Af*), and, to a lesser extent, *C. albicans* all demonstrated no discernible MIC or breakpoint when treated with FLC up to 128 μg/mL. The MIC of FLC against *Cn* MRL862, a resistant strain, was observed at 32 μg/mL, 16-fold higher than the wild-type strain H99; this MIC was 4-fold lower than the reported value of 128 μg/mL, suggesting a reduction of heteroresistance due to serial passage in drug-free media.^43,44^*Cn cdc50*Δ was notably more sensitive to azoles than was H99. The MICs of FLC and ITR against *cdc50*Δ *w*ere 0.125 and 0.002 μg/mL, respectively. Similar to the susceptibility change of AMB against *Cg cdc50*Δ compared to its wild-type, we observed the MIC of AMB was unchanged between *Cn cdc50*Δ and H99 at 0.5 μg/mL.^18^ MICs of CAS against *Cn* strains were consistent with reported values ranging from 4 to 16 μg/mL depending on the strain.^23,35^

**Table 1:** MICs of Key Compounds against Various Strains Analyzed in This Study Incubated in RPMI1640 at 37 °C for 48 h^a^

species	strain	FLC	ITR	AMB	CAS	KKOO	KKOO-NH_2_
C. neoformans	H99	2	0.125	0.5	16	16	8
C. neoformans	MRL862	32	0.031	0.125	16	8	8
C. neoformans	*cdc50*Δ	0.125	0.002	0.5	4	4	4
C. neoformans	*apt1*Δ	0.5	0.5	0.5	8	4	4
C. neoformans	*apt2*Δ	2	0.125	0.25	16	8	4
C. neoformans	*apt3*Δ	2	0.062	0.5	16	8	4
C. neoformans	*apt4*Δ	2	0.062	0.25	16	8	4
C. neoformans	*crm1*Δ	2	0.062	0.5	16	8	4
C. neoformans	Crm1^OE^	0.25	0.016	0.5	16	8	4
C. glabrata	2001	8	0.5	1	0.5	16	8
C. glabrata	FKS1-S629P	2	0.125	1	2	16	8
C. glabrata	FKS2-S663P	2	0.125	1	4	16	8
C. albicans	SC5413	>16	>1	1	0.25	32	16
C. auris	B11245	>128	0.5	1	1*	>128	16
A. fumigatus	AF293	>128	0.125	1	0.125*	>128	32

a * = Paradoxical growth observed.

The KKOO peptide possessed MICs of 16 μg/mL against wild-type *Cn* H99 and *C. glabrata* strain 2001, 32 μg/mL against *C. albicans* Sc5314. On its own, KKOO possessed MICs against *C. auris* B11245 and *A. fumigatus* AF293 > 128 μg/mL by 48 h. These results show that substituting lysine with ornithine greatly increased the activity of AW9-Ma against *Cn*, and imparted measurable MICs against several *Candida* spp. The double ornithine substituted version of the peptide, KKOO was called “Cryptomycin” since it is a synthetic peptide inspired by a protein sequence in *Cn* and it synergizes with several common antifungals against multiple fungal pathogens. Drug synergy between the original KKOO peptide and the standard antifungal compounds are reported in the Supporting Information, Tables S2–S5. Subsequently, the C′ amidated version of the peptide, KKOO-NH_2_, was identified in a larger screen of derivatives and called Cryptomycinamide. KKOO-NH_2_ demonstrated MICs of 8 μg/mL against *C. neoformans* H99, MRL862, and *C. glabrata* 2001, FKS1, and FKS2, 16 μg/mL against *C. albicans* SC5413, *C. auris* B11245, and 32 μg/mL against *A. fumigatus* AF293, at 48 h. This MIC data indicates that the C′ amidation markedly improved the antifungal activity of the KKOO peptide.

### Cryptomycinamide Exhibits Broad Spectrum Antifungal Drug Synergy with Multiple Antifungals

With initial MICs recorded, the next set of experiments endeavored to assess the potential drug synergy between the peptide and commonly used antifungals FLC, ITR, AMB, and CAS. FLC activity was indifferent to peptide exposure; no changes in MIC were observed as a result of peptide exposure against the strains examined in this study, Table 2. Combinations of KKOO-NH_2_ with either ITR or AMB demonstrated drug synergy, FIC index <0.5 against *C. neoformans* H99 and FLC-resistant strain MRL862, Tables 3, and 4, respectively. Notably, in the presence of 2 μg/mL KKOO-NH_2_, 4-fold less than the peptide MIC, the MIC of ITR was reduced from 0.125 by a factor of 8 to 0.016 μg/mL, Table 3. Similarly, the MIC of AMB was reduced 8-fold from 0.5 to 0.062 μg/mL in the presence of 1 μg/mL KKOO-NH_2_, a factor of 8 less than the MIC, against *Cn* H99 at 48 h, Table 4. We believe that the difference in drug synergy between the peptide and ITR versus FLC combinations was due to the structural differences of the azole; ITR possesses an alkyl tail missing in FLC that may help it cross the PM in the presence of the peptide.

**Table 2:** Drug Synergy between KKOO-NH_2_ and FLC in RPMI1640, 37 °C for 48 h^a^

species	MIC of KKOO-NH_2_ (μg/mL)	concentration of KKOO-NH_2_ (μg/mL) supplemented in RPMI media				
		0	1	2	4	8
C. neoformans H99	8	2	2	2	1	
*Cn* MRL862	8	32	32	32	32	
C. glabrata 2001	8	8	8	8	8	
*Cg* FKS1 S629P	8	2	2	2	1	
*Cg* FKS2 S663P	8	0.5	0.5	0.5	0.5	
C. albicans SC5413 *	16	>16	>16	>16	>16	
C. auris B11245	16	>128	>128	>128	>128	
A. fumigatus AF293	32	>128	>128	>128	>128	>128

a Tabulated data represents the new MIC of FLC (μg/mL) in combination with the peptide. * = the MICs of azoles against *C. albicans* could not be determined due to the persistence of an intermediate phenotype, Supporting Information, Figure S2.

**Table 3:** Drug Synergy between KKOO-NH_2_ and ITR in RPMI1640, 37 °C for 48 h^a^

species	MIC of KKOO-NH_2_ (μg/mL)	concentration of KKOO-NH_2_ (μg/mL) supplemented in RPMI media				
		0	1	2	4	8
C. neoformans H99	8	0.125	0.031	0.016	0.008	
*Cn* MRL862	8	0.062	0.062	0.016	0.004	
C. glabrata 2001	8	0.5	0.25	0.125	0.031	
*Cg* FKS1 S629P	8	0.062	0.031	0.016	0.008	
*Cg* FKS2 S663P	8	0.031	0.016	0.004	0.002	
C. albicans SC5413 *	16	>1	>1	>1	>1	
C. auris B11245	16	0.5	0.125	0.062	0.016	
A. fumigatus AF293	32	0.125	0.125	0.062	0.062	0.031

a Tabulated data represents the new MIC of ITR (μg/mL) in combination with the peptide. * = the MICs of azoles against *C. albicans* could not be determined due to the persistence of an intermediate phenotype, Supporting Information, Figure S2.

**Table 4:** Drug Synergy between KKOO-NH_2_ and AMB in RPMI1640, 37 °C for 48 h^a^

species	MIC of KKOO-NH_2_ (μg/mL)	concentration of KKOO-NH_2_ (μg/mL) supplemented in RPMI media				
		0	1	2	4	8
C. neoformans H99	8	0.5	0.062	0.062	0.031	
*Cn* MRL862	8	0.25	0.125	0.031	0.016	
C. glabrata 2001	8	1	0.5	0.25	0.25	
*Cg* FKS1 S629P	8	0.5	0.25	0.125	0.062	
*Cg* FKS2 S663P	8	0.25	0.25	0.062	0.016	
C. albicans SC5413	16	1	0.5	0.25	0.25	
C. auris B11245	16	1	0.5	0.5	0.25	
A. fumigatus AF293	32	1	0.5	0.25	0.5	0.5

a Tabulated data represents the new MIC of AMB (μg/mL) in combination with the peptide.

KKOO-NH_2_ sensitized multiple *Candida* spp., including *auris*, *albicans*, and *glabrata*, to CAS, FIC index <0.5, Table 5. Notably, in the presence of 2 μg/mL KKOO-NH_2_, 8-fold less than the MIC, the MIC of CAS was reduced 8-fold from 1 to 0.125 μg/mL against *C. auris* at 48 h. Interestingly, peptide treatment hypersensitized *C. glabrata* FKS1 S629P, but only mildly sensitized FKS2 S663P, to CAS treatment. In the presence of 2 μg/mL KKOO-NH_2_, 4-fold less than the peptide MIC against all three *C. glabrata* strains examined here, the MIC of CAS was reduced by 16-fold against *Cg* FKS1 from 1 to 0.062 μg/mL and reduced 4-fold against *Cg* FKS2 from 4 to 1 μg/mL. We determined that FKS2 demonstrated a relative susceptibility change most similar to the wild-type *Cg* 2001 of the two transformants since a 4-fold reduction of CAS MIC was also observed in *Cg* 2001. The reason why peptide exposure led to such drastically different relative CAS sensitivity changes of *Cg* FKS1 versus *Cg* 2001 or FKS2 strains is yet to be determined.

**Table 5:** Drug Synergy between KKOO-NH_2_ and CAS in RPMI1640, 37 °C for 48 h^a^

species	MIC of KKOO-NH_2_ (μg/mL)	concentration of KKOO-NH_2_ (μg/mL) supplemented in RPMI media				
		0	1	2	4	8
C. neoformans H99	8	16	16	16	4	
*Cn* MRL862	8	16	16	8	4	
C. glabrata 2001	8	0.5	0.5	0.125	0.004	
*Cg* FKS1 S629P	8	1	0.5	0.062	0.004	
*Cg* FKS2 S663P	8	4	2	1	1	
C. albicans SC5413	16	0.25	0.5	0.062	0.008	
C. auris B11245 **	16	1	0.5	0.125	0.031	
A. fumigatus AF293 **	32	0.125	0.031	0.031	0.031	0.031

a Tabulated data represents the new MIC of CAS (μg/mL) in combination with the peptide. ** = at 48 h, a paradoxical growth effect was observed for these strains when treated with CAS; the MIC was recorded as the well exhibiting less than 20% growth compared to the positive control.

**Figure 1:**
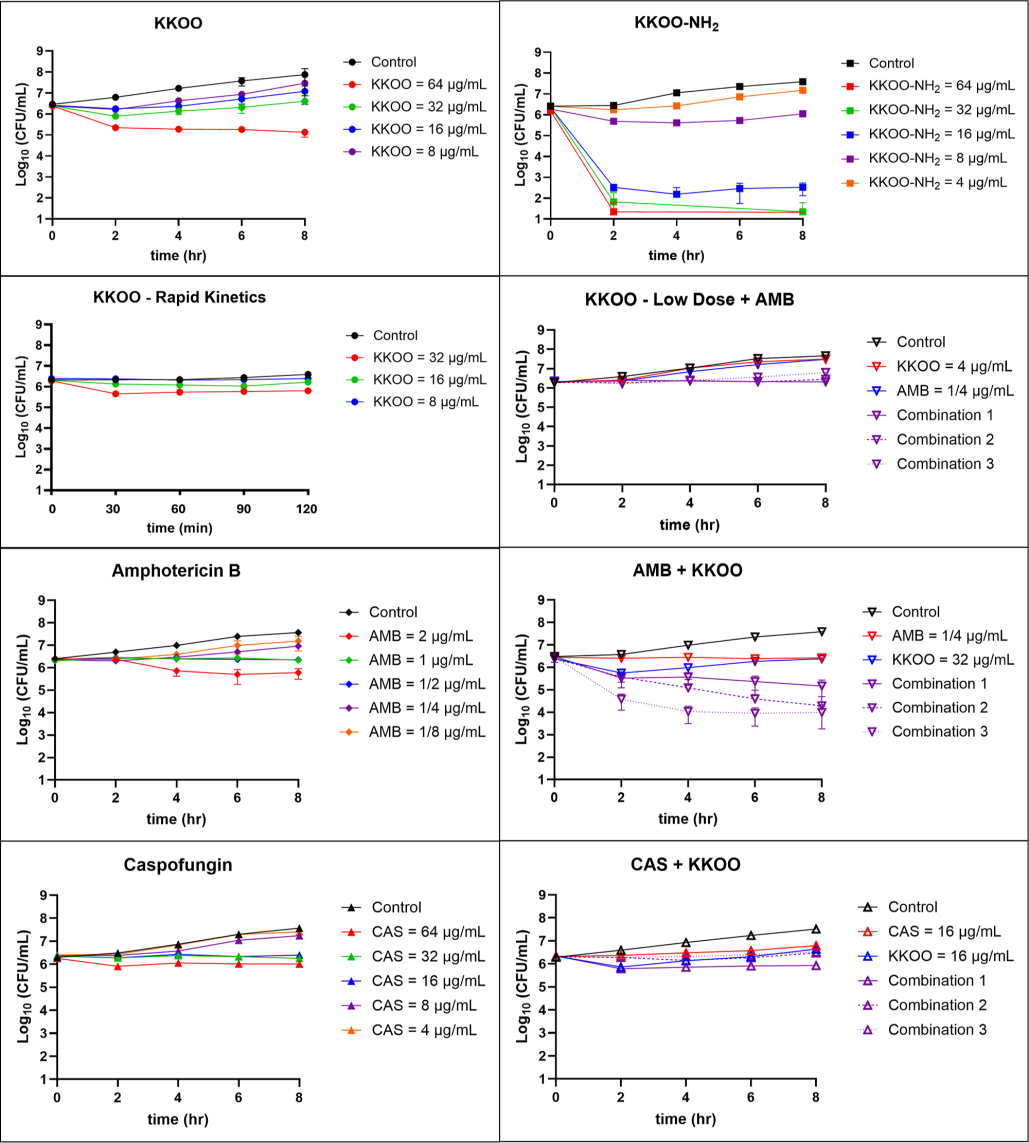
Killing kinetics of KKOO, KKOO-NH_2_, CAS, AMB, and antifungal drug combinations with KKOO against *C. neoformans* H99. Two million cfu/mL were inoculated in RPMI1640, dosed with peptide, and incubated at 30 °C, 225 rpm. Experiments were conducted with three biological replicates, and representative datasets were chosen. Error bars represent ±1 standard deviation between technical replicates. Error bars not shown were smaller than the marker used to represent the data point. KKOO + AMB low dose plot: combinations 1–3 were dosed with KKOO at 4 μg/mL and titrated with decreasing AMB concentrations at 0.25, 0.125, and 0.062 μg/mL, respectively. AMB + KKOO plot: combinations 1–3 were dosed with 0.25 μg/mL AMB and titrated with decreasing concentrations of KKOO at 32, 16, and 8 μg/mL. CAS + KKOO plot: combinations 1–3 were dosed with 16 μg/mL CAS and titrated with decreasing concentrations of KKOO between 16, 8, and 4 μg/mL, respectively.

We observed that KKOO on its own was somewhat fungicidal based on the reduced cfu following 2× MIC peptide treatment. However, KKOO demonstrated a short half-life in the kinetic killing assay, with peak killing activity by 2 h and subsequent rebound of the population through 8 h, Figure 1. KKOO-NH_2_ possesses superior fungicidal activity to its progenitor KKOO and can kill >99.9% of *Cryptococcus* cells at 2× MIC by 2 h, with no rebound by 8 h. These results suggest that KKOO could have been degraded or deactivated by an excreted carboxypeptidase that does not degrade KKOO-NH_2_ as efficiently.^45,46^ Alternatively, it is possible that C′ amidation increased the binding efficiency of the peptide to its essential target. Since KKOO was an early lead, we proceeded with proof-of-concept experiments to understand the kinetics of peptide combinations of KKOO with antifungals AMB and CAS. Combinations of KKOO and CAS possessed an additive effect of the early killing effect of the peptide followed by the prolonged fungistatic effect of CAS against *Cn*. Combinations of KKOO and AMB were rapidly fungicidal at higher concentrations and were still fungistatic at lower concentrations.

### Peptide Localizes to the PM, Stimulates ROS Production, and Leads to Intracellular Calcium Spike

**Figure 2:**
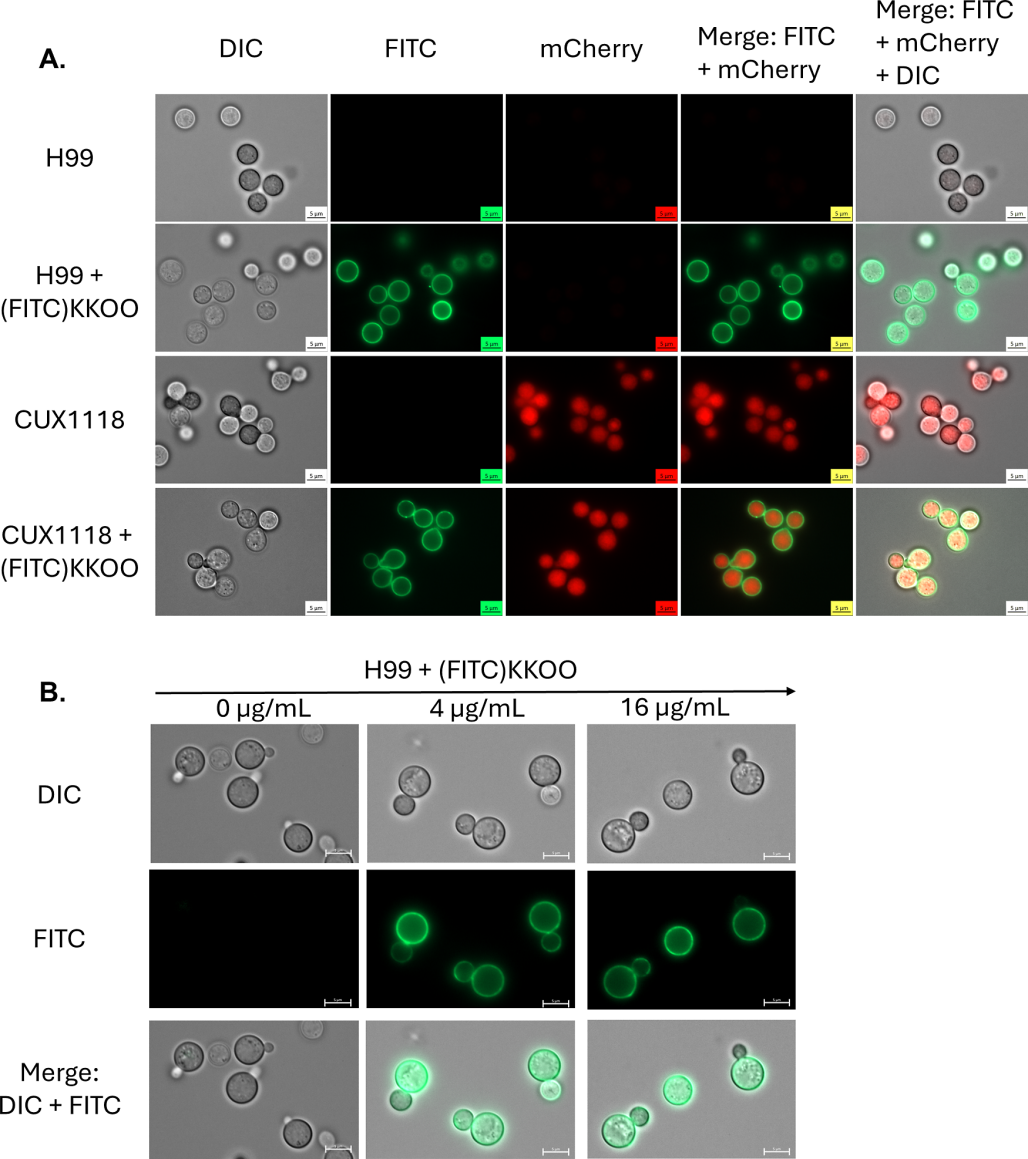
PM localization of a fluorescently labeled version of KKOO, (FITC)KKOO. (A) (FITC)KKOO localizes to the PM in both wild-type H99 as well as CUX1118, a *cdc50*Δ strain expressing cytosolic mCherry under a β-actin promoter. (B) H99 titrated with (FITC)KKOO. These experiments were conducted with a single biological replicate. Multiple images were recorded for each condition, and a representative image was chosen for publication.

A FITC labeled version of KKOO, “Myristic acid {Dap(FITC)}WGGIOONYA”, ((FITC)KKOO) was synthesized in-house and used to check the localization in *Cryptococcus* cells via fluorescence microscopy, Figure 2. It was observed that the fluorescent signal was localized to the PM at all concentrations of peptide examined in this study. Drug synergy was assessed to determine if the (FITC)KKOO peptide still possessed activity close to the unlabeled version, Supporting Information, Table S6. (FITC)KKOO was able to sensitize *Cn* H99 to ITR and AMB. In the presence of 4 μg/mL (FITC)KKOO, the MIC of AMB was reduced by a factor of 4 from 0.5 to 0.125 μg/mL, FIC index <0.5, against *Cn* H99. Persistence of drug synergy between (FITC)KKOO and both AMB and ITR at these peptide concentrations suggests that PM localization is likely a real phenomenon exhibited by the unlabeled version of the peptide as well.

**Figure 3:**
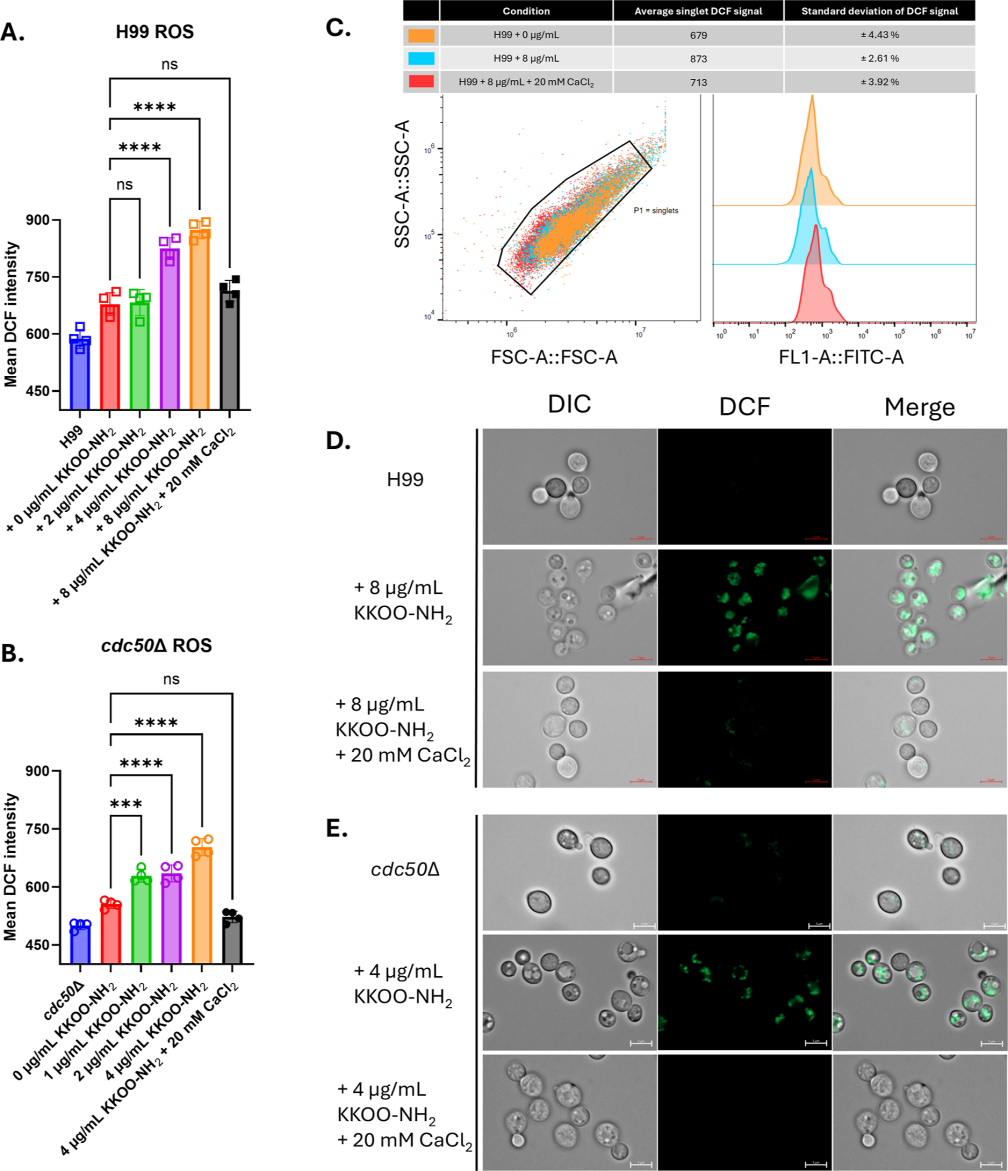
Intracellular ROS production detected by the H_2_DCFDA indicator. (A,B) Quantification data of ROS levels in H99 (A) and *cdc50*Δ (B), as a function of KKOO-NH_2_, concentration in the absence or presence of added extracellular calcium. Statistical significance resulted from a one-way ANOVA with multiple comparisons to a control group, ****P < 0.0001, ***P = 0.0001. Error bars represent ±1 standard deviation between the four technical replicates. (C) Gating strategy of flow cytometry measurements; first, FSC-A × SSC-A were gated for singlets. Next, a histogram of number of events as a function of fluorescence signal intensity of singlets was recorded. Mean signal intensity data for each sample was tabulated and plotted in (A,B). (D) Fluorescence microscopy images of peptide-treated H99 with DCF fluorescence. (E) Representative fluorescence microscopy images of peptide-treated *cdc50*Δ. These experiments were conducted with two biological replicates, a representative dataset was chosen.

ROS production and Ca^2+^ signaling have been reported as a coordinated physiological stress response in numerous organisms.^47−49^ Our previous study showed that *cdc50*Δ is hypersensitive to CAS treatment compared to wild-type H99 and its unregulated, high intracellular Ca^2+^ level in response to CAS treatment contributes to cell death, measured by an increase in ROS production.^35^ To understand the potential mechanism of fungal killing by peptide treatment, we measured the ROS production and intracellular calcium signaling following KKOO-NH_2_ treatment. ROS production after 30 min of peptide exposure was measured using the H_2_DCFDA indicator. Upon exposure to intracellular ROS, a metabolite of H_2_DCFDA chemically transforms into a fluorescent molecule, 2-(2,7-dichloro-6-hydroxy-3-oxo-3*H*-xanthen-9-yl)benzoic acid (DCF), which possesses excitation and emission spectra similar to those of FITC. Elevated ROS production was observed in *Cn* H99 and *cdc50*Δ treated with KKOO-NH_2_ as low as 4 and 1 μg/mL, respectively. When incubated in calcium-enriched YPD, no ROS increase was detected in both strain backgrounds treated with peptide at their MIC, as shown in Figure 3. These results indicate that peptide treatment led to stress-induced cell death, which can be reversed by calcium supplement.

**Figure 4:**
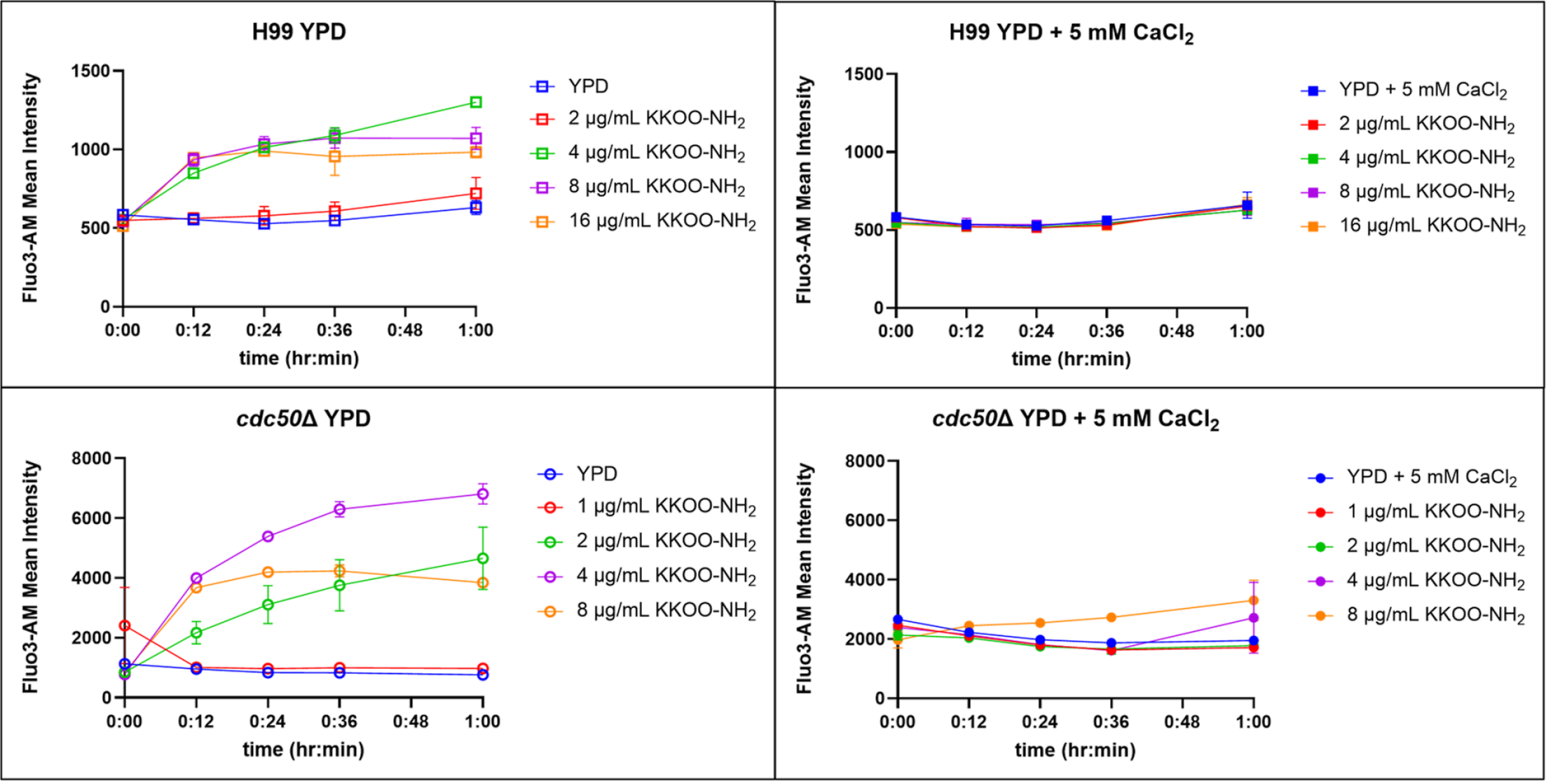
Calcium supplement desensitizes the peptide-mediated fungal killing in both wild-type and *cdc50*Δ. Intracellular calcium levels were determined by measuring the Fluo-3AM signal intensity in *C. neoformans* H99 and *cdc50*Δ in YPD treated with different levels of peptide KKOO-NH_2_, with or without calcium supplement, *n* = 3 technical replicates, 100,000 events collected per replicate on the flow cytometer. Error bars in each plot represent ±1 standard deviation between technical replicates. These experiments were conducted with two biological replicates, a representative dataset was chosen. Error bars indicate standard deviation.

Similarly to ROS measurements, intracellular calcium signaling was measured using a flow cytometer after staining cells with a calcium sensitive fluorescent molecular probe, Fluo-3AM, and treating them with KKOO-NH_2_ in YPD, Figure 4. Differences in intracellular calcium signaling between treated and untreated cells were observed within 12 min of peptide exposure and remained elevated for the 60 min time course. Like the observations in the ROS experiment, intracellular calcium increase was not observed in peptide-treated cells incubated in calcium-enriched YPD. This result indicates that intracellular calcium level regulation to maintain calcium homeostasis is an important mechanism of fungal cell tolerance to the peptide-induced stress response, which is consistent with previous studies.^35^

### Peptide Activity Is Calcium-Dependent

Previous studies have shown that modifying microbiological media with salts such as CaCl_2_ can have a drastic effect on the growth of fungi and their responses to antifungals.^19,35^ In the case of CaCl_2_, the divalent cation, Ca^2+^, is involved in fungal cell stress response. To understand how the peptide activity may involve calcium and stress response, the MICs of KKOO, KKOO-NH_2_, FLC, and CAS were assessed as a function of the calcium concentration in liquid YPD media. FLC activity was mostly indifferent to enriched calcium conditions, as shown in Table 6. It was observed that calcium enrichment completely desensitized the wild-type strain to KKOO and KKOO-NH_2_, Tables 7 and 8, respectively. Effects of calcium enrichment on the susceptibility of *Cryptococcus* to CAS similar to literature values were recorded, Supporting Information, Table S7.^35^

**Table 6:** Effect of Calcium on the MIC of FLC in μg/mL, 37 °C, 48 h^a^

	concentration of Ca^2+^ (mM) supplemented in YPD				
*Cn* strain	0	2.5	5	10	20
H99	8	8	8	8	8
*cdc50*Δ	0.5	0.5	0.5	0.25	0.25
*apt1*Δ	0.25	0.5	1	0.5	0.5
*crm1*Δ	4	4	4	8	8
Crm1-oe	1	2	2	2	2

a Calcium-dependent MIC assays were completed with two technical replicates and one biological replicate each.

**Table 7:** Effect of Calcium on the MIC of KKOO in μg/mL, 37 °C, 48 h

	concentration of Ca^2+^ (mM) supplemented in YPD				
*Cn* strain	0	2.5	5	10	20
H99	32	>128	>128	>128	>128
*cdc50*Δ	4	8	8	8	8
*apt1*Δ	8	32	128	128	>128
*crm1*Δ	8	>128	>128	>128	>128
Crm1-oe	8	>128	>128	>128	>128

**Table 8:** Effect of Calcium on the MIC of KKOO-NH_2_ in μg/mL, 37 °C, 48 h

	concentration of Ca^2+^ (mM) supplemented in YPD				
*Cn* strain	0	2.5	5	10	20
H99	8	128	>128	>128	>128
*cdc50*Δ	2	4	8	8	4
*apt1*Δ	4	16	64	64	128
*crm1*Δ	4	128	>128	>128	>128
Crm1-oe	4	128	>128	>128	>128

The MIC of KKOO-NH_2_ increased 16-fold from 8 to 128 μg/mL when increasing calcium enrichment in YPD from +0 to +2.5 mM CaCl_2_, and >128 μg/mL in YPD enriched with ≥5 mM CaCl_2_, Table 8. This marked decrease in sensitivity to the peptide was observed in other calcium homeostasis mutants, such as *crm1*Δ and Crm1^oe^. Interestingly, *cdc50*Δ remained mostly susceptible to peptide treatment at all concentrations of calcium enrichment. The largest MIC deviation was a 4-fold increase from 2 to 8 μg/mL when increasing the calcium enrichment in YPD from +0 to +5 mM CaCl_2_. This observation means that the calcium-dependent susceptibility of *Cn* to the peptide is itself mediated by Cdc50, which suggests Cdc50 coordinates PM-based Ca^2+^ homeostasis as previously hypothesized.^35^

In order to control for the effects of enriching YPD with a divalent cation, MICs and ROS production were also measured as a function of the MgCl_2_ supplement at the same millimolar concentrations of cation as in the CaCl_2_-modified experiments. The MIC of KKOO-NH_2_ against *Cn* H99 increased 4-fold from 8 to 32 μg/mL when increasing the magnesium enrichment in YPD from +0 to +20 mM MgCl_2_, Supporting Information, Table S8. No peptide susceptibility changes were observed against *Cn cdc50*Δ in magnesium-enriched media, Table S9. ROS production in H99 induced by peptide exposure was inhibited after 30 min of treatment in the magnesium-enriched condition, similar to the levels observed in the calcium-enriched media at 16 μg/mL KKOO-NH_2_, Figure S1. Taken together, the MIC variations and ROS production as a function of divalent cation enrichment in the media suggest there may be reduced binding efficiency of the peptide to its target by the presence of high levels of salt; however, only calcium enrichment was able to rescue cell viability, while magnesium enrichment failed to induce cells to overcome stress-induced cell death as indicated by the 48 h MICs in these media. The link between peptide activity and calcium homeostasis may be vital in understanding precisely how the peptide kills fungal cells and requires more research to elucidate.

Among the four lipid flippases (Apt1–4) in *Cn*, Apt1 has been reported to be important for stress tolerance, virulence, and polysaccharide secretions.^22,50,51^ We tested *Cn apt1*Δ and found that it demonstrated an intermediate decrease in sensitivity to KKOO-NH_2_ between *cdc50*Δ and H99, showing some MIC increase directly proportional to calcium concentration, increasing 32-fold from 4 to 128 μg/mL when increasing the calcium enrichment from +0 to +20 mM CaCl_2_. The intermediate susceptibility changes of KKOO and KKOO-NH_2_ observed as a function of the extracellular calcium concentration against *apt1*Δ but not the *Crm1* mutants suggest that Apt1 plays a role in peptide activity. The KKOO and KKOO-NH_2_ peptides were inspired by a Cdc50 sequence that targets the Cdc50-ATPase interaction by design. However, various *Cn* flippase knockouts demonstrated increased sensitivity to the peptide, Table 1, indicating that it likely also interacts with additional targets that remain to be identified. Furthermore, while lipid flippases are important for drug resistance and pathogenicity, they are nonessential in *C. neoformans*.

### Apt1–Cdc50 Possesses Unique Interactions between Key Cdc50 Residues and ATPase, Providing a Potential Conserved Drug Target and Binding Mode

An analysis of the genome revealed that there are four lipid flippases in *C. neoformans*.^26^ The first three, Apt1, Apt2, and Apt3 are classified as P4A, and Apt4 is classified as P4B.^52^ Given that the peptide localizes to the PM, Apt2 localizes to intracellular compartments, and Apt4 functions independently of Cdc50,^52,53^ we hypothesized that the peptide might interrupt native interactions between Cdc50 and the ATPase in either the wild-type Apt1–Cdc50 or Apt3–Cdc50 complex. AlphaFold 2 was utilized in multimer mode to predict protein structures of the ATPase–Cdc50 complexes, and the structures were analyzed using PyMoL (https://www.pymol.org/).

**Figure 5:**
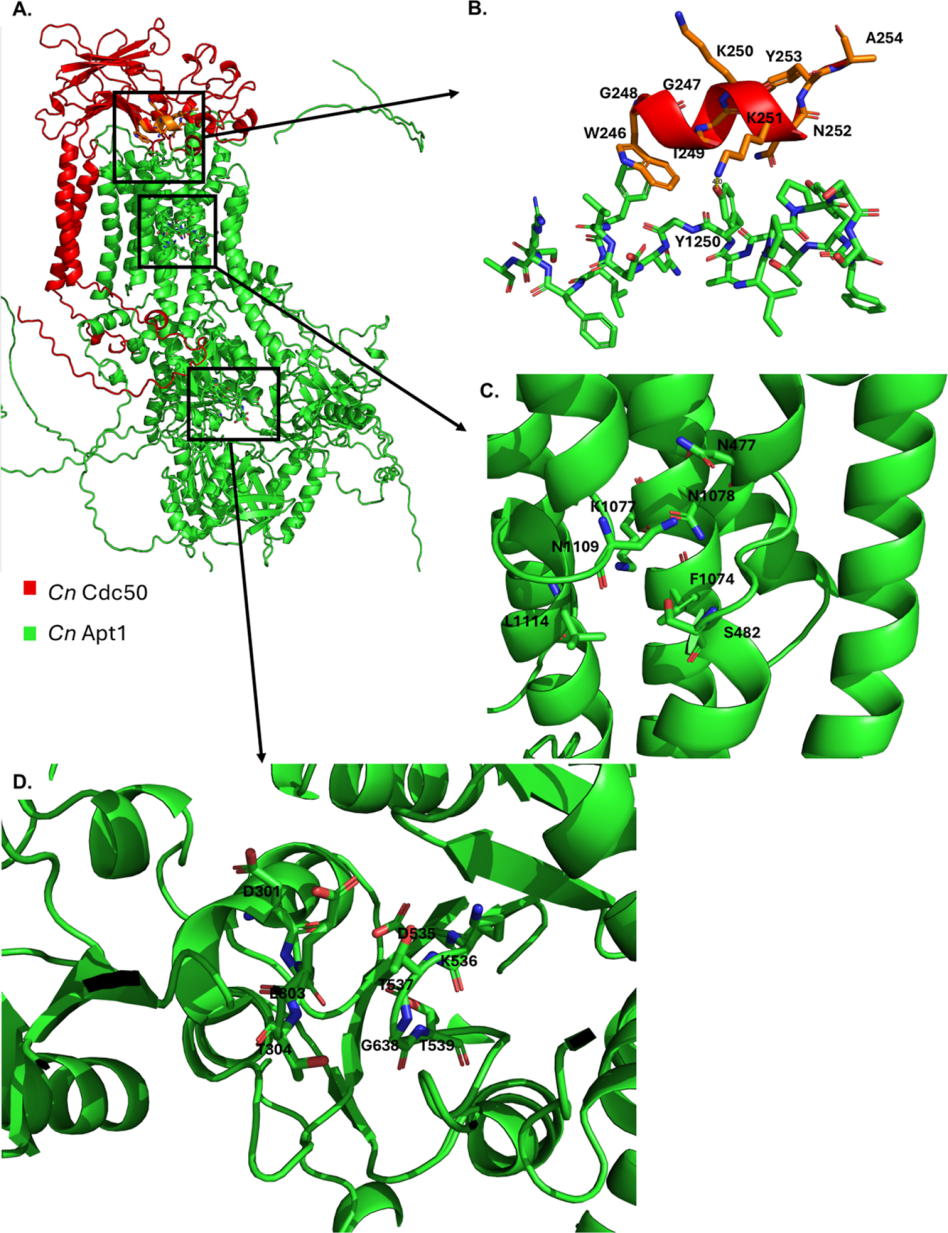
Structural prediction of Cdc50 K251 interaction with*Cn*Apt1 Y1250. (A) Full image of the predicted α–β heterodimeric structure of Apt1–Cdc50 determine by AlphaFold2. (B) Highlighting the contact between the two subunits of the complex Apt1–Cdc50 along the extracellular PM interface, where the peptide progenitor sequence is located. (C) Residues of the lipid-binding pocket as predicted by a sequence alignment with known lipid bound flippase structures. (D) Conserved “DKTGT” phosphorylation (P) domain and “DGET” dephosphorylation (A) domain.

It was observed that *Cn* Cdc50 K251 makes close contact, within 4 Å, of the side chain hydroxyl group of *Cn* Apt1, Y1250, Figure 5. A similar analysis was carried out on the Apt3–Cdc50 complex, Supporting Information, Figure S3; however, no such interactions were observed between the Cdc50 K250/K251 residues and the ATPase. Interestingly, the contact between K251 and Y1250 could provide a posthoc rationalization as to why the single ornithine-substituted peptide, K6O, had the most significant improvement compared to its related derivative K5O in the original substitution screen of AW9-Ma, Table S1. Since the sixth amino acid of the peptide “K6” corresponds with Cdc50 K251, and since ornithine possesses a smaller side chain similar in length to lysine and is a more polar source of a cationic charge than lysine, it potentially could competitively inhibit the native Cdc50 interaction with Apt1 at this site. Furthermore, other SAR screen data support this notion as well because further reduction of the cationic residue side chain in the Dab- and Dap-substituted peptides attenuates peptide activity, Supporting Information, Table S1, while ornithine-substituted peptides exhibit optimized activity. When considered together with the calcium-dependent and strain-dependent MICs, localization studies, and additional biophysical characterization like the vesicle leakage assay, Figure 8A, it is more likely that a specific peptide–protein interaction is occurring than a simple surfactant-like or pore forming effect.

**Figure 6:**
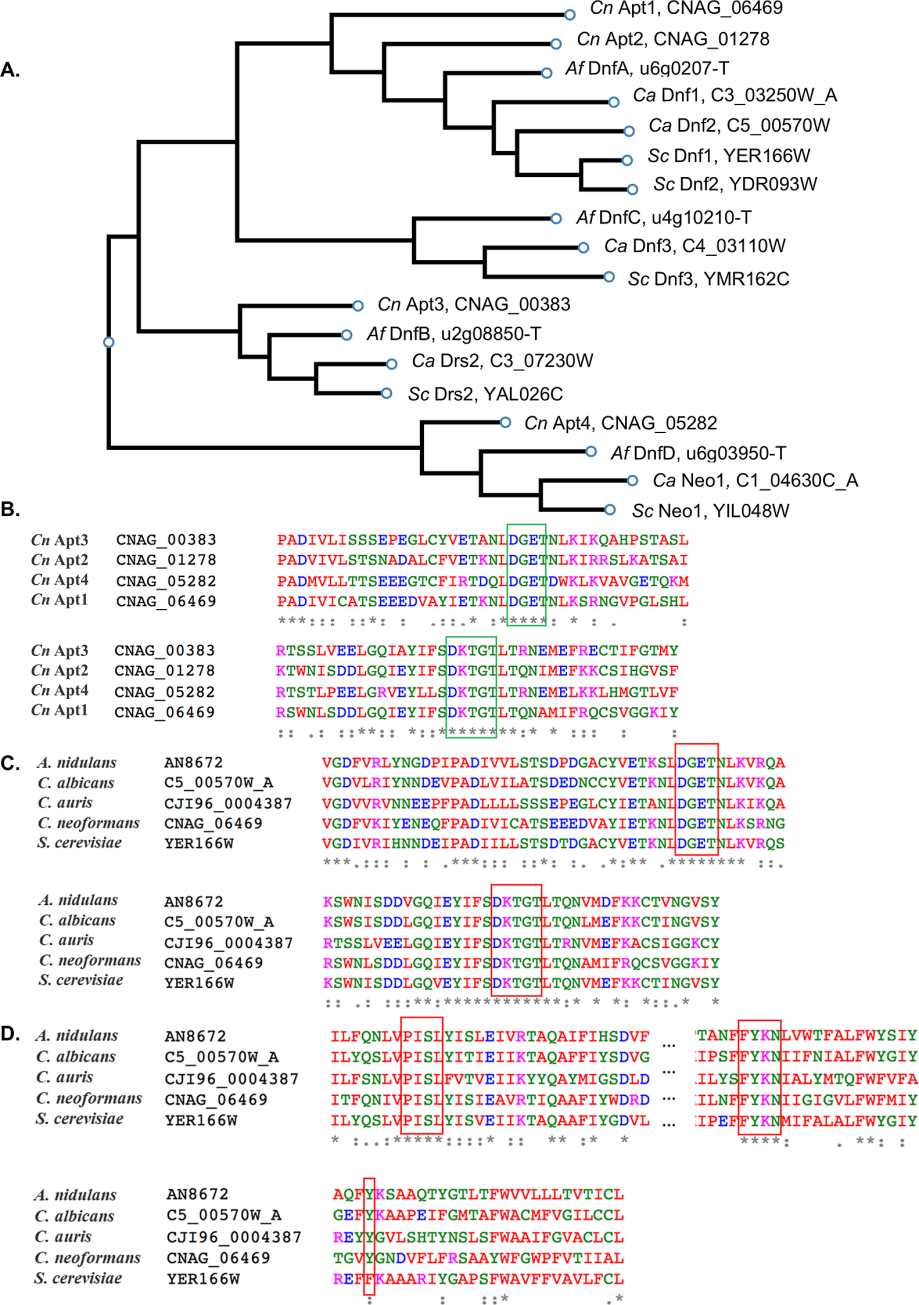
Phylogeny and sequence alignment of various P4 ATPases in model fungal organisms. (A) Phylogenetic tree of P4 ATPase flippases in *Cryptococcus* and other model fungal organisms using the multiple sequence alignment (ClustalW) method. (B) Identification of phosphorylation “DKTGT” and dephosphorylation “DGET” sites of the Apt1–4 in *C. neoformans*. (C) Identification of phosphorylation “DKTGT” and dephosphorylation “DGET” sites of the Apt1 homologues between species. (D) Identification of important highly conserved sequences of fungal lipid flippases. Notably “PISL”, a key phospholipid-binding site, “FYKN”, a conserved motif in the central core of a TM domain of the ATPase, and “*Cn* Apt1 Y1250”, a highly conserved residue that may be involved in peptide binding.

Since flippases are conserved in fungi, we wanted to understand if homology between the different flippases could help to rationalize the improved broad-spectrum activity of the ornithine-substituted peptides. First, a phylogenetic tree was produced to analyze the potential relationship among the various known lipid flippases in model fungi. We noted that *Cn* Apt1 was closely related to *Saccharomyces cerevisiae* (*Sc*) Dnf1, *Ca* Dnf1, and *Af* DnfA, Figure 6A.^52^ Based on the interesting AlphaFold2 structural prediction from the Apt1–Cdc50 protein complex, Figure 5, we found it suggestive that related homologues in other species are also involved in peptide activity when it was observed that Y1250 in *Cn* Apt1 is conserved among its homologues in other fungal pathogen species, Figure 6D, providing a potential conserved target and binding mode. This notion is supported by our MIC data against different fungal pathogens. However, further investigation is warranted to confirm these results experimentally.

### Peptide Treatment Sensitizes *Cryptococcus* to Macrophage Killing after 24 h Coincubation

**Figure 7:**
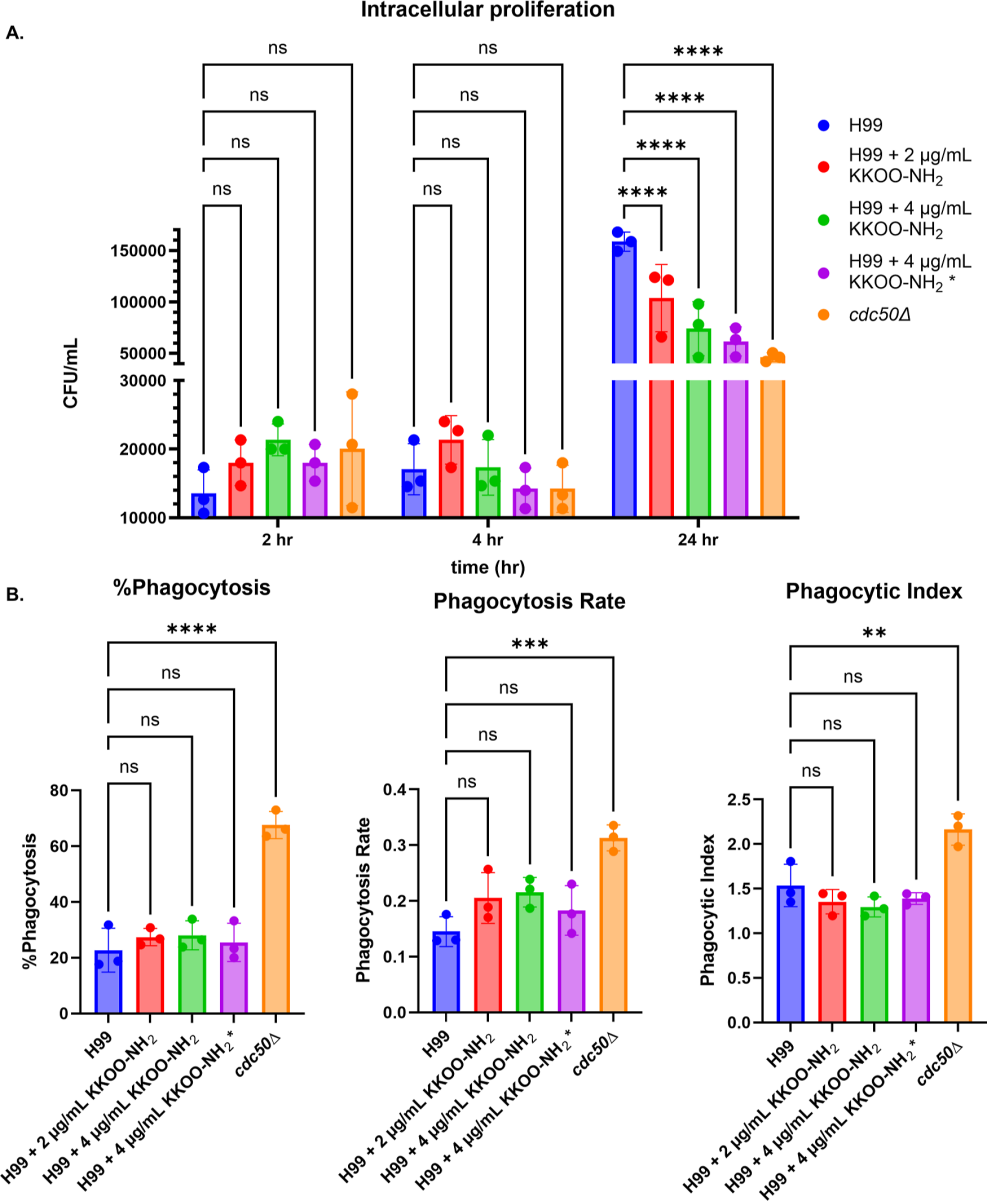
Macrophage killing assay demonstrates increased macrophage killing of peptide-treated yeast cells. (A) Yeast cells pretreated with the peptide KKOO-NH_2_ were cocultured with macrophage-like cell line J774, and extracellular yeast cells were carefully washed away after 2 h of coincubation. Fungal cfus were determined at 2, 4, and 24 h postcoculturing. Two-way ANOVA was computed comparing each condition to a control within each time point, ****P < 0.0001. (B) Phagocytosis assay of pretreated yeast cells coincubated with macrophages. One-way ANOVAs were computed for each quantity measured, % phagocytosis, phagocytosis rate, and phagocytic index, ****P < 0.0001, ***P = 0.0005, **P = 0.0021. Condition 4, * = this condition was prepared with twice the operating cfu/mL to control for the number of live cells that the macrophages are coincubated with. These assays were conducted with three technical replicates and multiple biological replicates. Representative biological replicate was chosen to present the data. Error bars represent ±1 standard deviation between technical replicates. Statistical tests for A and B were computed using GraphPad PRISM, version 8.0, α = 0.05, *n* = 3.

Yeast cells were treated with KKOO-NH_2_ at sub-MIC for 30 min, and then the cells were opsonized and coincubated with macrophage (Φ) J774 cells for 2, 4, and 24 h. No statistically significant difference in fungal proliferation inside Φ was detected at 2 or 4 h. However, by 24 h, there were statistically significant differences between the untreated control and the treated groups, Figure 7A, *P* < 0.0001, α = 0.05, *n* = 3, two-way ANOVA, simple effects within rows where rows were time points and columns were treatment conditions. No statistically significant differences in phagocytic index or phagocytosis rate were detected for sub-MIC peptide-treated yeast cells coincubated with Φ for 2 h, Figure 7B. The lack of differences in the phagocytosis assay provides validation for the Φ killing assay since the reduced cfu in the killing assay was not due to a defect in yeast cell uptake of the Φ caused by exposing yeast cells to the peptide at sub-MIC levels.

**Figure 8:**
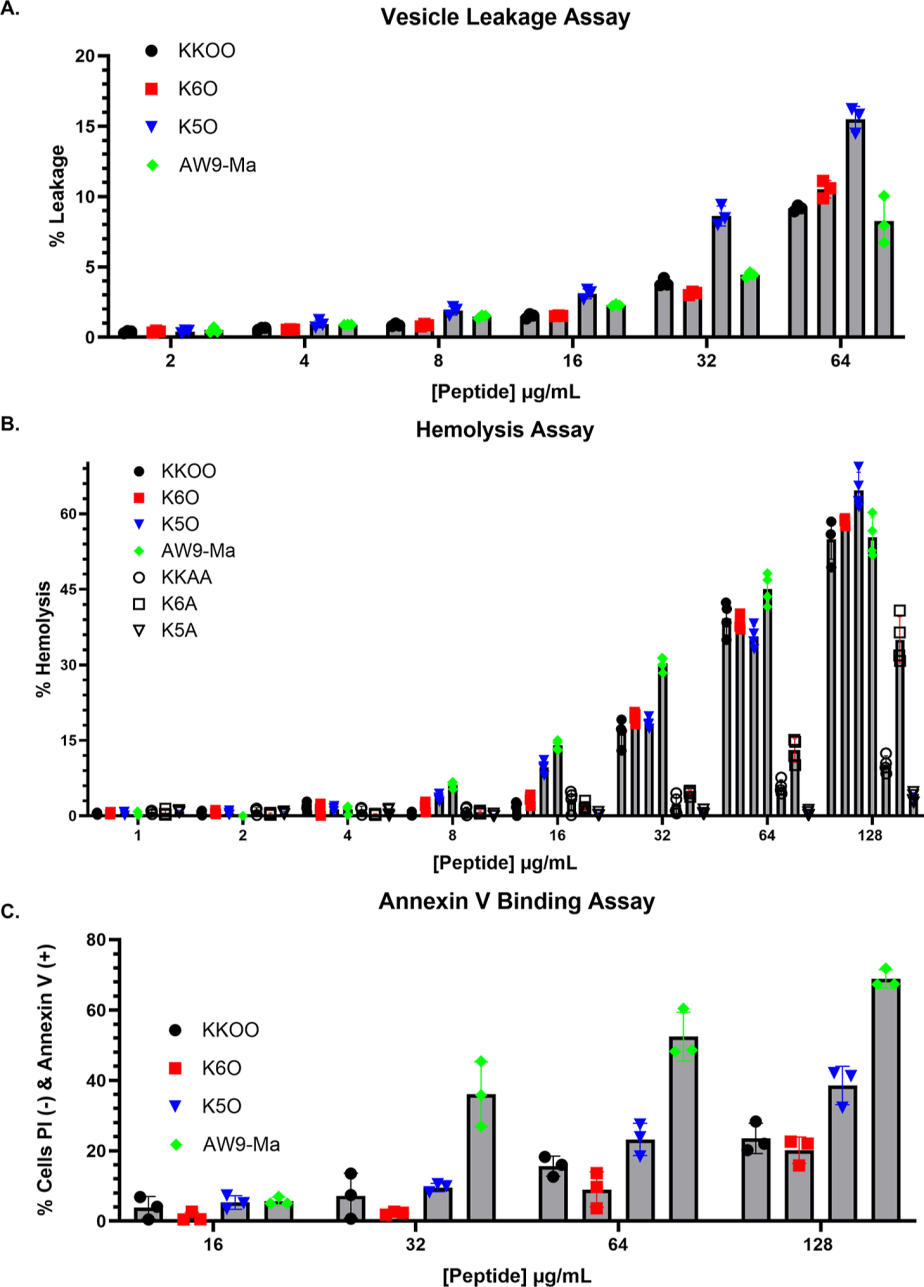
Characterization of the potential cytotoxicity of the developed peptides. (A) Listed peptides were coincubated with PC vesicles in a vesicle leakage assay, *n* = 3. (B) Listed peptides were coincubated with human RBCs in hemolysis assay, *n* = 4. (C) Annexin V-binding assay of the listed peptides coincubated with *Cn* H99 cells in PBS, *n* = 3. Baseline measurement for untreated cells. These three experiments were conducted with a single biological replicate. In each of these experiments, the error bars represent ±1 standard deviation.

We performed hemolysis assays using human red blood cells (RBCs) and vesicle leakage assays using phosphatidylcholine (PC) vesicles to determine the potential cytotoxicity of our peptides. These additional experiments suggest that the ornithine-substituted peptides are less toxic than the original AW9-Ma peptide, Figure 8B, evident by the low leakage percentage and low hemolysis under 32 μg/mL. Annexin V-binding assays, Figure 8C, did show an increase of externalized phosphatidylserine (PS) at higher peptide concentrations than MIC. It should only be expected that a significant annexin V signal increase over untreated control would occur at sub-MIC if the peptide inhibited the principal PS flippase in *Cn*.

Since Drs2 is the primary PS flippase in *Sc*, and Apt3 in *Cn* has the highest homology to Drs2 of the four lipid flippases in *Cn*, Figure 6A, it is reasonable to hypothesize that Apt3 could also be the primary PS flippase in *Cn*. Prior studies suggested that phosphatidylethanolamine (PE) is a ligand of Apt3 by utilizing NBD-labeled lipid uptake and retention in a heterologous expression system of Apt proteins in a flippase-deficient strain of *Sc*.^53^ Additional validation is required to confirm the substrate of Apt3 using either *C. neoformans* knockouts or by using a chemical assay with purified flippase proteins in vitro. Due to the substrate specificity problem of lipid flippases, more experiments are required to definitively determine if PS located in the PM is regulated by Apt3 in *Cn*. If Apt3 is the primary regulator of PS in the PM, then one would only expect to see a substantial Annexin V signal at sub-MIC if the peptide targeted Apt3. Since Apt1 is the flippase of broad substrate specificity in *Cn*, inhibiting it alone is unlikely sufficient to lead to substantial changes in PS localization but instead a more general loss of lipid asymmetry between the two leaflets of the PM.^52^

### Altering PM Lipid Distribution May Be the Source of Antifungal Drug Synergy with Flippase Inhibitors

Previous studies have demonstrated that ergosterol distribution between the two sides of the PM was much closer to equilibrium in flippase knockout strains than in the wild-type strains, suggesting a link between ergosterol localization and PM lipid distribution, which is regulated by flippases.^54^ Ergosterol affinity of each lipid depends on the degree of unsaturation of the FAT and the type of polar headgroup. Ergosterol tends to colocalize with saturated fatty acids in the PM with large head groups like sphingolipids, PC, and PS, predominantly on the cytoplasmic leaflet of the PM.^54,55^ Drug synergy between the peptides and AMB could arise if the peptide inhibits a protein responsible for maintaining the lipid microenvironment (e.g., lipid flippase), which in turn alters ergosterol distribution and localization on the PM.^56^ Ergosterol is essential in fungi, and inhibiting proper localization of ergosterol could be a stressor-sensitizing fungal cells to AMB’s effects. Lipid flippase function has been reported to be required for proper ergosterol and PM protein trafficking through the ER-Golgi apparatus, and *cdc50*Δ may have a disruption of the ergosterol trafficking to the proper PM location, leading to increased sensitivity against antifungal compounds.^57−61^ This intracellular function of flippases may contribute to the additional sensitivity of *cdc50*Δ to our peptides.

**Figure 9:**
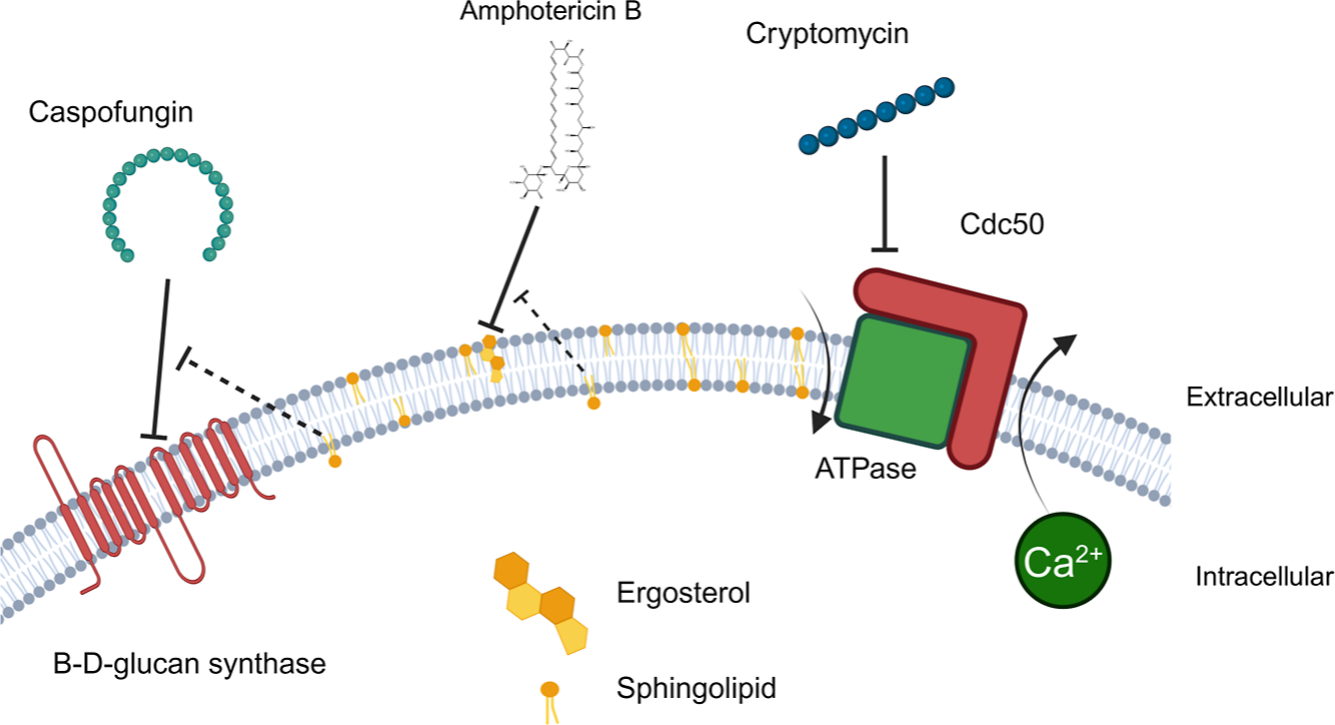
Working model of how the peptide might sensitize fungi to commonly used antifungals. If the peptide inhibits ATPase–Cdc50 located on the PM, it could influence either PM lipid distribution or calcium efflux from the cell, or possibly both. Altering the lipid microenvironment could potentially sensitize fungi to AMB through interactions between ergosterol and sphingolipids. Similarly, *Candida* spp. may have been sensitized to CAS through a related mechanism because echinocandin interaction with FKS is modulated by sphingolipids in *C. glabrata*. Precisely how Cdc50 coordinates Ca^2+^ homeostasis across the PM is yet to be determined: does Cdc50 efflux Ca^2+^ directly or indirectly by forming a complex with a PM calcium channel-like the intracellular Cdc50-Crm1 complex in the ER? This figure was prepared using BioRender (Toronto, CA).

In the case of *C. glabrata*, sphingolipids were shown to modulate the interaction between echinocandins and β-d-glucan synthase (FKS).^17^ Alterations to the lipid microenvironment of the PM likely resulted from the peptide inhibiting native interactions between Cdc50 and the ATPase of broad substrate specificity, reproducing the previously reported CAS susceptibilities of *Cn*, *Ca*, and *Cg cdc50*Δ mutants through a related mechanism.^18,19,23^ Based on the data presented here and previous studies published by multiple groups, we summarize our working model of peptide activity in Figure 9 to explain these potential interactions.

## Conclusions

This report details the development of Cryptomycin and its more potent derivative Cryptomycinamide. These peptides, particularly the latter, are promising lead compounds that exhibit broad spectrum antifungal drug synergies against multiple species of fungal pathogens. The peptide was observed to localize to the PM. Peptide exposure promotes fungal ROS production, stimulates intracellular calcium signaling, and sensitizes fungal cells against macrophage killing. Experiments conducted in calcium-enriched media demonstrate that these processes are likely mediated through changes in intracellular calcium concentrations that result from Cdc50-dependent interactions. The peptide possesses promising drug synergy in combination with existing therapeutics by sensitizing fungi to their activity, possibly by altering the lipid microenvironment on the PM via inhibiting the lipid flippase of broad substrate specificity. Investigating the peptide’s utility in vivo is ongoing and pending formulation into a stable delivery vehicle.

## Methods and Materials

### Materials

#### Chemicals

The peptide sequences examined in this study are reported in Table 9. Initial peptide screening of KKOO was carried out with peptides manufactured in-house at Seton Hall University. The alanine substituted peptides were purchased from Biomatik (Onterio, Canada). Additional peptides investigating SAR and peptide batches of >25 mg were purchased from GenScript (Piscataway, NJ). ITR was purchased from Selleckchem.com. FLC was purchased from Tokyo Chemical Industry (TCI) (Portland, OR). AMB was purchased from Bio Basic (Markham, Ontario, Canada). CAS was purchased from Ambeed (Arlington Heights, Il). All other chemicals were purchased from Sigma-Aldrich (St. Louis, MO).

**Table 9:** Peptide Sequences Examined in This Study^a^

peptide	sequence, N′ → C′	N′	C′	pred. MW	source
K5O	WGGIOKNYA	Myr	COOH	1232.54	A
K6O	WGGIKONYA	Myr	COOH	1232.54	A
KKOO	WGGIOONYA	Myr	COOH	1218.51	A, C
KKOO-NH_2_	WGGIOONYA	Myr	NH_2_	1217.53	C
KKOO-NHMe	WGGIOONYA	Myr	NHMe	1231.55	C
K5,6 Dab	WGGI{Dab}{Dab}NYA	Myr	COOH	1190.46	C
K5,6 Dap	WGGI{Dap}{Dap}NYA	Myr	COOH	1162.40	C
K5A	WGGIAKNYA	Myr	COOH	1189.47	B
K6A	WGGIKANYA	Myr	COOH	1189.47	B
KKAA	WGGIAANYA	Myr	COOH	1132.37	B
A9a	WGGIOONY{d-Ala}	Myr	COOH	1218.51	C
Y8y	WGGIOON{d-Tyr}A	Myr	COOH	1218.51	C
N7n	WGGIOO{d-Asn}YA	Myr	COOH	1218.51	C
O6o	WGGIO{d-Orn}NYA	Myr	COOH	1218.51	C
O5o	WGGI{d-Orn}ONYA	Myr	COOH	1218.51	C
I4i	WGG{d-Ile}OONYA	Myr	COOH	1218.51	C
W1w	{d-Trp}GGIOONYA	Myr	COOH	1218.51	C
G2HYP	W{Hyp}GIOONYA	Myr	NH_2_	1273.59	C
(FITC)KKOO	{Dap(FITC)} WGGIOONYA	Myr	COOH	1693.98	A

a Peptide sources: A = synthesized in-house at Seton Hall University; B = Biomatik (ON, CA); C = GenScript (Piscataway, NJ). O = l-ornithine, Dab = diaminobutyric acid, Dap = diaminopropionic acid, and Hyp = hydroxyproline. Predicted molecular weights calculated using ChemDraw, PerkinElmer (Waltham, MA).

#### Strains and Cell Lines

The fungal strains examined in this study are listed in Table 10. Human RBCs were provided by Interstate Blood Bank Inc. (IBBI) from a Diagnostic Red Blood Cell group of blood type O+. Donor blood was tested for pathogens by (IBBI) following FDA regulations before shipping. Murine macrophage cell line J774.16 was from ATCC (ATCC# TIB-67), and its stock was kept in liquid nitrogen before activation. Stock yeast cells were stored at −80 °C in 20% v/v glycerol in liquid YPD. Yeast stocks were thawed and spread onto YPD plates and incubated at 30 °C for 2 days, followed by subsequent passages on selection media. Cells were passaged biweekly on YPD plates to maintain healthy colonies for experiments and stored at 4 °C. Cells were sampled from YPD plates and cultured overnight at 30 °C, 225 rpm, in 5 mL of liquid YPD for assays the subsequent day. *C. auris* was unique because it was prepared for experiments by passaging overnight in 4 mL of liquid YPD + 10% m/V NaCl, at 30 °C.

**Table 10:** Fungal Strains Used in This Study

species	strain	selection marker	source
C. neoformans	H99		Perfect 1993^62^
*Cn*	MRL862		Sionov 2012^43^
*Cn*	*cdc50*Δ	G418	Xue lab^23^
*Cn*	*apt1*Δ	G418	Xue lab
*Cn*	*apt2*Δ	G418	Xue lab
*Cn*	*apt3*Δ	G418	Xue lab
*Cn*	*apt4*Δ	G418	Xue lab
*Cn*	*crm1*Δ	Nat	Xue lab^35^
*Cn*	Crm1oe	Nat	Xue lab^35^
*Cn*	CUX 1118-mCherry	G418	Xue lab
C. auris	B11245		CDC
C. albicans (*Ca*)	SC5314		ATCC
C. glabrata (*Cg*)	2001		ATCC
*Cg*	FKS1-S629P		Healey lab^63^
*Cg*	FKS2-S663P		Healey lab^63^
A. fumigatus (*Af*)	AF293		Rivera lab

#### Media

Liquid YPD media was prepared by adding 10 g/L yeast extract, 20 g/L peptone, and 100 mL/L of 20% w/v glucose solution to Milli-Q water to total 1 L. The mixture was autoclaved and allowed to cool for use. YPD plates were prepared similarly to liquid YPD, except with the addition of 20 g/L agar. Agar media was cooled at room temperature for at least an hour while being stirred before adding selection markers as needed. RPMI1640 media with and without phenol red were used. Premixed powder was purchased from Sigma and diluted with MilliQ water. MOPS was added at a concentration of 36.7 g/L, and a pH probe was used to adjust the pH to 7.0 with 1 M NaOH. The volume was adjusted to 1 L and filtered through a sterile 0.2 μ filter.

Figure preparation and statistical analysis. All figures and statistical tests were produced using GraphPad Prism version 8.0 (Boston, MA) unless otherwise specified. α values of 0.05 were used for all ANOVAs.

### Methods

#### Solid Phase Peptide Synthesis

In-house synthesis microwave (CEM, Matthews NC)-assisted solid phase peptide synthesis utilizing FMOC/*t*Bu protected amino acids (Advanced ChemTech, Louisville KY) was used to synthesize KKOO, K6O, and K5O at the 0.1 mmol scale. Wang resin was allowed to swell in DMF for several minutes prior to coupling the first amino acid. The first amino acid was loaded using quadruple coupling for 5 min using mol ratios of 5:8:5:1, FMOC-AA/DIEA/HCTU/peptide in DMF. Subsequent amino acid couplings used 3:6:3:1 mol ratios, respectively. The resin was rinsed twice with DMF after each amino acid coupling and the FMOC deprotection reaction. Myristic acid coupling reaction was run in duplicate to ensure quantitative labeling of the N′ terminus of the peptide using mole ratios of 1.5:3:1 myristic anhydride/DIEA/peptide for 5 min. Prior to cleavage of the peptide off of solid support beads were rinsed twice each with 5 mL of DMF, followed by 5 mL of MeOH, and finally 5 mL of DCM. The peptides were cleaved from the resin and deprotected using a mixture of 2.5% v/v H_2_O, 2.5% v/v TES, and 95% v/v TFA. Crude peptide was recovered via lyophilization and purified via prep chromatography using a C18 column and acetonitrile gradient from 5 to 95% H_2_O/ACN mixture. Fractions containing the target at a sufficient purity were pooled and lyophilized with a small amount of acetic acid. The purified dry powder recovered from the lyophilizer was demonstrated to be >95% purity via HPLC at 220 nm, and the identity was also confirmed by mass spectrometry; KKOO prepared in house was also characterized by NMR. Chemical characterization of peptides is provided in the Supporting Information, Figures S4–S12.

#### MIC and FIC Index Assay

All microdilution assays with fungi were carried out in accordance with CLSI protocols from M27 for yeasts and M38 for filamentous fungi, respectively, unless otherwise specified.^64,65^ Briefly, 96-well plates were charged with RPMI1640 media, with operating volume in the first column, 200 μL, and half operating volume, 100 μL, in the remaining wells. Drug stock solution in DMSO was aliquoted into the first well in each row. Half of the volume in the dosed well was serially diluted 2-fold until column 11. Column 11 was used as the negative growth control, and column 12 was used as the positive growth control. ON yeast cultures in YPD were washed twice in DD-H_2_O, resuspended in PBS, and adjusted to 5000 cfu/mL in RPMI. The 96-well plate was inoculated with 100 μL aliquots of cell suspension per well. Plates were incubated at 37 °C for 2 days.

To assess drug synergy, the initial media charged to the plate contained the peptide at twice the final operating concentration; the remaining processing steps were identical to the MIC experiment. Upon addition of 100 μL of inoculant, the final operating concentration was achieved. Control MIC plates were prepared alongside drug synergy plates to account for the day-to-day variability in any individual MIC. FIC index is computed using eq 1. All MIC assays and drug synergy assays were completed with two technical replicates per 96-well plate and a minimum of two biological replicates per drug combination. If a difference in MIC between the two technical replicates on the plate were observed, only the higher concentration was reported as the MIC to reduce the possibility of type 1 error (false positive)
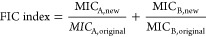
1

#### Kinetic Killing Assay

Culture tubes were prepared with aliquots of 2.0 mL of RPMI1640 (containing Q, MOPS, pH = 7, no phenol red). The tubes were sealed to prevent evaporation and were equilibrated with the incubation chamber overnight, at 30 °C. On the day of the experiment, overnight yeast cultures in YPD were washed twice with DD-H_2_O and resuspended in DD-H_2_O. The OD_600_ of the cell suspension was adjusted to 4.6, ≈2 × 10^6^ cfu/mL, in DD-H_2_O. A 200 μL aliquot of adjusted cell suspension was added systematically to each tube, followed by 200 μL sampling for the 0 h time point. Immediately after sampling, the antifungal compounds were added, and the tubes were placed in the incubator at 30 °C with shaking at 225 rpm. The sampled aliquot was transferred to a 96-well plate and serially diluted six times, 10-fold each time. The resulting cell suspensions were spotted onto YPD plates in a grid pattern of 5 μL per spot. Subsequently, tubes were briefly removed from the incubator, and 100 μL aliquots were sampled at 2, 4, 6, and 8 h time points. Cells were counted on the YPD plates at either 24 or 48 h after plate spotting, depending on when the colonies were countable. Images of the YPD plates were recorded on the Bio-Rad Gel imager after 48 h of incubation at 30 °C. Each condition was prepared in triplicate, and each sample was spotted on the YPD plate in triplicate. The concentration of yeast cells in colony forming units (cfu)/mL is computed using eq 2
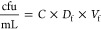
2*C* = number of colonies counted, *D*_f_ = dilution factor = 10^*n*–1^, *n* = column that the cells were counted, and *V*_f_ = volume factor = 1/0.005 mL.

#### Fluorescence Microscopy to Detect Peptide Cellular Localization

Overnight yeast cultures in YPD were washed twice with DD-H_2_O, resuspended in PBS, and adjusted to 2 × 10^7^ cfu/mL. In a 1.5 mL Eppendorf tube, 100 μL cell suspension was stained with FITC-KKOO at 128 μg/mL. The cells were stained for minutes at room temperature on a shaker at 200 rpm. The stained cells were recovered and fixed for 5 min at room temperature in 4% formaldehyde in PBS. Fixed cells were resuspended in a small amount of 1% formaldehyde in PBS for imaging. A glass slide was prepared with a 7.5 μL aliquot of the final cell suspension for imaging with a 100× objective using immersion oil. A Zeiss (Jena, Germany) Axioscope 5 equipped with an Axiocam mono 705 camera was used to image the slides. The software provided by the instrument manufacturer was used to process the images. The acquisition and gating parameters used to record and process the fluorescent microscopy images in Figure 2a are reported in Table 11.

**Table 11:** Operating Parameters Used for Fluorescence Microscopy Imaging and Data Processing for Figure 2A

parameter	channel		
	DIC	FITC	mCherry
% intensity	1.64	30	60
exposure time (ms)	1.8	50	200
white gate	4666	1227	1509
black gate	2618	192	100
gamma	1–1.5	1–1.5	1–1.5

#### Bioinformatics—AlphaFold2, Phylogeny, and Sequence Alignment

Alignment and phylogenetic reconstructions were performed using the function “build” of ETE3 3.1.2, as implemented on the GenomeNet (https://www.genome.jp/tools/ete/).^66^ User provided the ClustalW multiple sequence alignment. The tree was constructed using fasttree with slow NNI and MLACC = 3 (to make the maximum-likelihood NNIs more exhaustive).^67^ Values at nodes are SH-like local support. The three-dimensional structure of α–β heterodimeric complexes of *Cn* Apt1–Cdc50 and Apt3–Cdc50 were predicted using AlphaFold 2 through the online Cosmic 2 platform (https://cosmic-cryoem.org/tools/alphafold2/).^68^ Structures were analyzed, and images were recorded of the proteins using PyMoL software (Schrödinger, NY, NY).

#### Intracellular ROS Detection

Protocol was carried out in accordance with previously reported methodologies.^35^ Yeast cells were grown ON in liquid YPD, washed once in H_2_O, and resuspended in fresh YPD. The OD_600_ was adjusted to 0.2 in YPD. Cells were incubated for 3 h at 30 °C, 225 rpm, and then stained with 10 μM H_2_DCFDA for 2 h at 30 °C, 225 rpm, in the dark. Stained cells were washed twice with DD-H_2_O and resuspended in the minimal amount of YPD or calcium-enriched YPD needed to inoculate each technical replicate with cells. Each condition was prepared with four technical replicates. In the dark, the stained cells were titrated with KKOO-NH_2_ from 0.25 to 1× MIC and incubated for 30 min at 30 °C, 225 rpm, in 1 mL of YPD in 12 mL of vented culture tubes. After treatment, cells were transferred to 1.5 mL Eppendorf and centrifuged for 5 min at 2000 rpm. The cells were washed once with 0.5 mL of PBS and resuspended in 0.2 mL of PBS, and stored on ice for live-cell flow analysis, BD FACS Via (Franklyn Lakes, NJ). 100,000 events were recorded, and singlets were gated from SSC-A × FSC-A plot. Mean fluorescence signal intensity from the probe was plotted as a function of peptide concentration. Cells that remained after FACS were pooled based on their condition and imaged under the fluorescence microscope, Zeiss Axioscope 5.

#### Intracellular Calcium Measurement

Intracellular calcium measurement was carried out in accordance with previously reported methodologies.^35^ Yeast cells were sub cultured ON in YPD, and the next day cells were washed twice in D-Hanks (0.4 g/Liter KCl, 0.06 g/Liter KH_2_PO_4_, 8.0 g/Liter NaCl, 0.35 g/Liter NaHCO_3_, 0.06 g/Liter Na_2_HPO_4_·7H_2_O) and adjusted to 1 × 10^7^ cfu/mL in 5 mL D-Hanks. Fluo-3-AM stock solution in DMSO was diluted 1000-fold to operating stain concentration of 10 μM. Cells were stained in the dark for 30 min at 37 °C, 225 rpm, in a 12 mL culture tube. Stained cells were washed three times in D-Hanks and resuspended into a minimal amount of YPD or calcium-enriched YPD required to inoculate each condition. Each condition was prepared with three technical replicates. After inoculating 1 mL YPD with 0.2 mL cell suspension, 0.2 mL was aliquoted into a flow tube for the 0 min time point. The flow tube was placed on ice until flow analysis. The remaining cell suspension, 1 mL, was titrated with peptide from 0.25× to 2× MIC and incubated at 30 °C, 225 rpm, in the dark. To allow for sample processing for each time point, each set of replicates was inoculated and treated about 2 min apart. Subsequent 0.2 mL aliquots were drawn at 12, 24, 36, and 60 min for flow analysis. Live cells were analyzed using BD FACS via 100,000 events were collected, and singlets were gated for each sample. The mean fluorescence of the singlets was recorded and plotted as a function of peptide concentration.

### Phagocytosis Assay

#### Phagocytosis Assay Was Carried Out in Accordance with Previously Reported Protocols^23,69^

The J774A. I macrophage-like cell line (ATCC# TIB-67) was grown in DMEM supplemented with 10% FBS, 5% penstrep, and l-glutamine (1×), and passed 2–3 times per week. The cells were never used after 5 passages. Phagocytosis assay was performed in a 48-well plate using J774 cells (5 × 10^4^) in 0.5 mL of fresh DMEM were added to each well and incubated at 37 °C, 5% CO_2_. To activate the macrophages, 50 units/mL interferon γ (IFN-γ; Invitrogen) and 1 μg/mL lipopolysaccharide (LPS; Sigma) were added to each well. Yeast cells were adjusted to 2.4 × 10^6^ cfu/mL in RPMI1640. One mL of cell suspension was aliquoted into a 12 mL vented culture tube. The peptide was added to each tube, and the cells were incubated for 30 min at 30 °C, 225 rpm. The cells were pelleted and resuspended in 100 μL of 20% mouse compliment in PBS. The cells were opsonized at room temperature for 30 min. After opsonization, 10 μL of cell suspension was added to each well. Yeast cells were coincubated with macrophages for 2 h at 37 °C in 5% CO_2_ in technical triplicates per condition. After coincubation, the supernatant was removed, and the wells were washed 3 times with 200 μL DMEM + penstrep, and then fixed with 200 μL ice cold methanol for 30 min. The fixed cells were stained ON at 4 °C with Giemsa stain diluted 20-fold in PBS. The wells were washed twice with 200 μL PBS, and the macrophages were imaged using the Nikon Eclipse (Nikon, Japan) TS2 inverted microscope. The images were quantified using phagocytosis rate, % phagocytosis, and phagocytic index, eqs 3–5, between 200 and 300 macrophages were counted per technical replicate
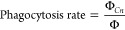
3
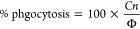
4
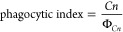
5Φ = total number of macrophages, Φ_*Cn*_ = total number of yeast positive macrophages, and *Cn* = number of phagocytosed yeast cells.

#### Macrophage Killing Assay

Macrophage killing assay was carried out in accordance with previously reported protocols.^23,69^ After the cell line was activated from −80 °C stock and passaged several times, the cells were washed and resuspended in fresh DMEM + FBS at 1 × 10^5^ cell/mL. Each well in a 3 column by 6 row grid in a 48-well plate was charged with 0.5 mL of cell suspension, approximately 50,000 cell/well. Macrophages were grown ON at 37 °C in a tissue culture incubator and were further activated with LPS. Yeast cells were grown ON using standard conditions. Fungal cells were washed twice with H_2_O, resuspended in PBS, and adjusted to 2.4 × 10^6^ cfu/mL in 5 mL RPMI. For each treatment condition, 1 mL of cell suspension was transferred to a 12 mL culture tube, titrated with KKOO-NH_2_ between 0.25× and 0.5× MIC, and incubated for 30 min at 30 °C at 225 rpm. Treated cells were transferred to a 1.5 mL Eppendorf tube, pelleted, and resuspended in 100 μL of 20% mouse compliment in PBS for opsonization. Prior to inoculating the wells with yeast cells for coincubation, the opsonized cells were diluted by an additional 20% with PBS. Each well was inoculated with 10 μL of cell suspension. Cocultures were prepared using three technical replicates. The 48-well plates were inoculated about 30 min apart to allow time to process each plate during the experiment. After 2 h of coincubation, each well was washed three times with 200 μL of warm DMEM + penstrep. After the final wash step, the wells were recharged with 0.5 mL of warm DMEM + FBS, and the plate was incubated for the remaining 2 or 22 h for the 4 and 24 h time points, respectively. After washing the 2 h plate with warm DMEM, the macrophages were lysed with 200 μL DD-H_2_O for 60 min. The resulting cell suspension was charged to a 96-well plate, serially diluted 10-fold, and spotted onto a YPD plate for colony counting. Plat spots for each technical replicate were prepared in triplicate to determine cfu concentration using eq 2.

#### Hemolysis Assay

The hemolysis assay was carried out in accordance with our previous report.^37^ Fresh RBCs were washed twice with PBS, resuspended, and adjusted to 5 × 10^8^ RBCs/mL in PBS. Drug dilutions were prepared in a 96-well plate similar to the MIC assays except PBS was the media, each peptide treatment condition consisted of four technical replicates, the operating volume was 100 μL, the negative control consisted of untreated RBCs in PBS, and the positive control consisted of RBCs lysed by 0.01% Triton X. RBCs were incubated with peptides for 1 h at 37 °C, then centrifuged at 1000 rpm for 5 min. Supernatant from each well was diluted 5-fold to 100 μL in fresh PBS. The absorbance of heme at 410 nm was measured in triplicate using a Spectra Max 340 plate reader (Molecular devices, Sunnyvale, CA). The hemolysis % was computed for each technical replicate using eq 6, where “*X*” is the absorbance of the sample, “*N*” is the average absorbance of the negative control, and “*P*” is the average absorbance of the positive control
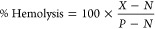
6

#### Vesicle Leakage Assay

Vesicle leakage assays were carried out in accordance with previously reported protocols.^70−72^ PC was resolubilized in 10 mM HEPES, 50 mM terbium chloride, and 100 mM sodium citrate. The lipids were extruded through a filter with a 100 nm pore size. The vesicles were purified using a Sephadex G-75 column and a running buffer of 10 mM HEPES and 300 mM sodium chloride. The opaque fractions were collected and pooled together for use in the assay. Peptides were dissolved in DMSO and serially diluted in 150 μM dipicolinic acid (DPA), 10 mM HEPES, and 300 mM sodium chloride in a 96-well plate to reach the final concentrations: 64, 32, 16, 8, 4, and 2 μg/mL. DMSO in the plates did not exceed 0.5%. Trace amounts of DMSO have been determined not to influence vesicle leakage.^73^

Lipids were then added to all the wells to give a lipid concentration of 0.5 mM PC. The peptide samples, positive controls (vesicles, DPA, and reduced Triton X), and negative controls (vesicles and DPA) were run in triplicate and excited at 270 nm and scanned for emission at 545 nm using a TECAN Spark plate reader. As the terbium–citrate complex leaks outside of the vesicles, the citrate exchanges with the DPA in the solution, causing the Tb^3+^–DPA complex to form, resulting in an increase in fluorescence. The results were reported as percent leakage using eq 7, in which “Fs” is the average fluorescence of the sample, “Fnc” is the fluorescence of the average of the negative controls, and “Fpc” is the fluorescence of the positive control. Samples were prepared in technical replicates of three
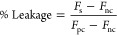
7

#### Annexin V-Binding Assay

The Annexin V-binding assay was carried out in accordance with our previous report.^37^ Each sample was prepared with three technical replicates of three. *C. neoformans* H99 cells were titrated with peptide from 16 to 128 μg/mL in PBS at 25 °C for 20 min. Simultaneously, cells were stained with viability indicator propidium iodide (PI) 2 μg/mL and Annexin V conjugated to Alexafluor350, diluted per the manufacturer’s recommended dilution factor. After the treatment period, cells were washed in 4% formaldehyde and resuspended in 1% formaldehyde in PBS for flow cytometry analysis, and 50,000 events were collected on the MACS Quant analyzer (Miltenyi Biotec, Auburn, CA). First, singlets were gated. Then, viable cells were gated from the singlets. Viable cells were gated for the correct morphology. Cells with the correct morphology were analyzed on the final plot, analyzing PI signal versus Annexin V signal. The percentages of cells in the population that were considered Annexin V positive and PI negative were recorded and plotted for each condition.

## Supporting Information Available

MICs of intermediate SAR screen peptides; FLC drug synergy with KKOO; ITR drug synergy with KKOO; AMB drug synergy with KKOO; CAS drug synergy with KKOO; antifungal drug synergy with (FITC)KKOO; effect of CaCl_2_ enrichment on CAS activity; effect of MgCl_2_ enrichment on antifungal activity against Cn H99; effect of MgCl_2_ enrichment on antifungal activity against Cn cdc50Δ; ROS production in CaCl_2_ and MgCl_2_-enriched medias; phenotype of *C. albicans* Sc5314 treated with azoles in an MIC assay; AlphaFold 2 model of Cn Apt3–Cdc50 complex; LCMS trace of KKOO-NH_2_; mass spectrum of main peak for KKOO-NH_2_; HPLC chromatogram of KKOO-NH2; HPLC mobile phase gradient; solid phase peptide synthesis microwave heating settings; tabulated material specifications from in-house KKOO; UV–vis absorbance of KKOO; HPLC traces + MSD slices of KKOO; NMR proton spectrum of KKOO; H NMR aliphatic expansion; H NMR aromatic expansion; and H–H COSY spectrum (PDF)

## List of Abbreviations

DCF: 2-(2,7-dichloro-6-hydroxy-3-oxo-3*H*-xanthen-9-yl)benzoic acid
ITR: itraconazole
Dab: l-diaminobutyric acid
Dap: l-diaminopropionic acid
ACN: acetonitrile
LPS: lipopolysaccharide
AMB: amphotericin B
Orn: l-ornithine
*Af*: *Aspergillus fumigatus*
Φ: macrophage
*Ca*: *Candida albicans*
MeOH: methanol
*Cg*: *Candida glabrata*
CAS: caspofungin
MIC: minimum inhibitory concentration
Cdc50: cell division cycle 50
CDC: Centers for Disease Control
Ma: myristic acid FAT
cfu: colony forming units
Myr: myristylated peptide FAT
*Cn*: *Cryptococcus neoformans*
HCTU: *O*-(1*H*-6-chlorobenzotriazole-1-)-1,1,3,3-tetramethyluronium
KKOO: cryptomycinyl
KKOO-NH_2_: cryptomycinamide hexafluorophosphate
DCM: dichloromethane
ON: overnight
DIEA: diisopropylethylamine
PBS: phosphate buffer solution
DMF: dimethylformamide
PC: phosphatidylcholine
DMSO: dimethyl sulfoxide
PE: phosphatidylethanolamine
DPA: dipicolinic acid
PS: phosphatidylserine
DD-H_2_O: double distilled water
PM: plasma membrane
DMEM: Dulbecco’s modified Eagle medium
Pi: propidium iodide
ROS: reactive oxygen species
FAT: fatty acid tail
RBCs: red blood cells
FBS: fetal bovine serum
RPMI: Roswell Park Memorial Institute
FACS: flow assisted cell sorting
RPM: rotations per minute
FLC: fluconazole
SAR: structure–activity relationship
FMOC: fluorenylmethoxycarbonyl
*Sc*: *Saccharomyces cerevisiae*
FITC: fluorescein isothiocyanate
TM: transmembrane
FIC: fractional inhibitory concentration
TES: triethylsilane
TFA: trifluoroacetic acid
IFN-γ: interferon
YPD: yeast peptone dextrose
