# Supplementary material for: Improved Broad Spectrum Antifungal Drug Synergies with Cryptomycin, a Cdc50-Inspired Antifungal Peptide
Source: ACS Infect Dis. 2024 Oct 29;10(11):3973–93. doi: 10.1021/acsinfecdis.4c00681 (PMC11555678; doi:10.1021/acsinfecdis.4c00681)

Supporting Information - Improved Broad Spectrum Antifungal Drug Synergies with Cryptomycin, a Cdc50 Inspired Antifungal Peptide

Robert J. Tancer[a], Siddhi Pawar[a], Yina Wang[a], Cristina R. Ventura[b], Gregory Wiedman[b*], Chaoyang Xue[a*]

[a] Public Health Research Institute and Department of Microbiology, Biochemistry, and Molecular Genetics, New Jersey Medical School, Rutgers University, Newark NJ 07103.

[b] Department of Chemistry and Biochemistry, Seton Hall University, South Orange NJ 07079.

* Corresponding authors

gregory.wiedman@shu.edu

xuech@njms.rutgers.edu

Microbiology

Table S1. MICs of SAR screen peptides against key strains examined in this study. * = insoluble in delivery vehicle, DMSO.

	C. neoformans		***C. glabrata***	***C. albicans***
**Peptide**	**H99**	***cdc50Δ***	**2001**	**Sc5413**
K5O	>128	8	>128	-
K6O	32	4	32	32
KKOO	16	4	16	16
KKOO-NH_2	8	4	8	16
KKOO-NHMe	8	4	16	16
K5,6Dab	>128	8	>128	>128
K5,6Dap	>128	>128	>128	>128
K5A	-	>128	>128	-
K6A	-	>128	>128	-
KKAA	-	>128	>128	-
A9a	>128	4	>128	>128
Y8y *	-	-	-	-
N7n	32	8	>128	>128
O6o	16	4	>128	>128
O5o	>128	8	>128	>128
I4i	16	4	64	>128
W1w	16	4	32	>128
G2HYP	8	4	16	16
(FITC)KKOO	>128	128	-	-

Antifungal drug synergies with KKOO

Table S2. Fluconazole (FLC) drug synergy with KKOO. Drug synergy between KKOO and fluconazole in RPMI1640, 37°C for 48 hr. Tabulated data represents the new MIC of fluconazole (µg/mL) in combination with the peptide. * = MICs of azoles against *C. albicans* were unclear due to the observed intermediate phenotype, **Figure S2.**

Species	MIC of KKOO	Concentration of KKOO (µg/mL) supplemented in RPMI media				
		0	1	2	4	8
C. neoformans H99	16	2	2	2	2	1
Cn MRL862	8	32	64	32	32	-
C. glabrata 2001	16	8	8	8	4	4
Cg FKS1 S629P	16	2	2	2	2	-
Cg FKS2 S663P	8	0.5	0.5	0.5	0.5	-
C. albicans Sc5413*	32	>16	>16	>16	>16	0.125
C. auris B11245	>128	>128	>128	>128	>128	>128
A. fumigatus AF293	>128	>128	>128	>128	>128	>128

Table S3. Itraconazole (ITR) drug synergy with KKOO. Drug synergy between KKOO and fluconazole in RPMI1640, 37°C for 48 hr. Tabulated data represents the new MIC of fluconazole (µg/mL) in combination with the peptide. * = MICs of azoles against *C. albicans* were unclear due to the observed intermediate phenotype, **Figure S2.**

Species	MIC of KKOO	Concentration of KKOO (µg/mL) supplemented in RPMI media				
		0	1	2	4	8
C. neoformans H99	16	0.125	0.062	0.016	0.016	0.008
Cn MRL862	8	0.062	0.031	0.016	0.556	-
C. glabrata 2001	16	0.5	0.25	0.125	0.062	0.062
Cg FKS1 S629P	16	0.125	0.062	0.031	0.016	-
Cg FKS2 S663P	8	0.031	0.016	0.016	0.556	-
C. albicans Sc5413*	32	>1	>1	>1	>1	>1
C. auris B11245	>128	0.5	0.25	0.062	0.031	-
A. fumigatus AF293	>128	0.125	0.125	0.062	0.062	-

Table S4. Amphotericin B (AMB) drug synergy with KKOO. Drug synergy between KKOO and fluconazole in RPMI1640, 37°C for 48 hr. Tabulated data represents the new MIC of fluconazole (µg/mL) in combination with the peptide.

Species	MIC of KKOO	Concentration of KKOO (µg/mL) supplemented in RPMI media				
		0	1	2	4	8
C. neoformans H99	16	0.5	0.25	0.062	0.062	0.062
Cn MRL862	8	0.25	0.125	0.031	0.016	-
C. glabrata 2001	16	1	0.5	0.25	0.25	0.125
Cg FKS1 S629P	16	1	0.5	0.25	0.25	-
Cg FKS2 S663P	8	0.25	0.25	0.125	0.031	
C. albicans Sc5413	32	1	0.5	0.25	0.125	0.125
C. auris B11245	>128	1	0.5	0.5	0.25	-
A. fumigatus AF293	>128	1	0.25	0.25	0.25	

Table S5. Caspofungin (CAS) drug synergy with KKOO. Drug synergy between KKOO and fluconazole in RPMI1640, 37°C for 48 hr. Tabulated data represents the new MIC of fluconazole (µg/mL) in combination with the peptide. ** = at 48 hours, these strains demonstrated a marked paradoxical growth effect when treated with caspofungin, MIC was recorded if treatment resulted in less than 20% growth compared to the positive control at the reported concentration.

Species	MIC of KKOO (µg/mL)	Concentration of KKOO (µg/mL) supplemented in RPMI media				
		0	1	2	4	8
C. neoformans H99	16	16	16	16	16	8
Cn MRL862	8	16	16	16	8	-
C. glabrata 2001	16	0.5	0.5	0.25	0.016	0.556
Cg FKS1 S629P	16	1	0.5	0.125	0.008	-
Cg FKS2 S663P	8	4	2	2	1	
C. albicans Sc5413	32	0.25	0.5	0.125	0.556	0.008
C. auris B11245**	>128	1	1	0.25	0.031	-
A. fumigatus AF293**	>128	0.125	0.062	0.031	0.031	-

Table S6. Synergy between (FITC)KKOO and standard antifungals. *Cn* H99, RPMI1640, 37 °C, 72 hr data point.

	Concentration of (FITC)KKOO (µg/mL) supplemented in the media.		
	0	4	8
FLC	2	2	2
ITR	0.125	0.062	0.031
AMB	0.5	0.125	0.062
CAS	16	16	16

Table S7. Effect of Calcium on the MIC of CAS in µg/mL, 37 °C, 48hr

	Concentration of Ca^{2+} (mM) supplemented in YPD				
Cn strain	0	2.5	5	10	20
H99	8	16	32	64	128
cdc50Δ	4	8	16	32	8
apt1Δ	8	16	32	32	64
crm1Δ	8	32	32	64	>128
Crm1-oe	8	32	32	128	128

Table S8. Effect of Magnesium enrichment on the MICs of various antifungal compounds against *Cn* H99, 37 °C, 48hr.

	Concentration of Mg^{2+}(mM) supplemented in YPD				
	0	2.5	5	10	20
Flc	4	4	2	2	2
KKOO	16	32	32	32	128
-NH2	8	8	16	16	32
Cas	8	16	16	32	64

Table S9. Effect of Magnesium enrichment on the MICs of various antifungal compounds against *Cn cdc50Δ*, 37 °C, 48hr.

	Concentration of Mg^{2+}(mM) supplemented in YPD				
	0	2.5	5	10	20
Flc	½	¼	¼	1/8	1/16
KKOO	8	8	8	4	8
-NH2	4	4	4	4	4
Cas	4	8	8	8	8

Figure S1. ROS production controlled for divalent cation selection, Pep = KKOO-NH_2.

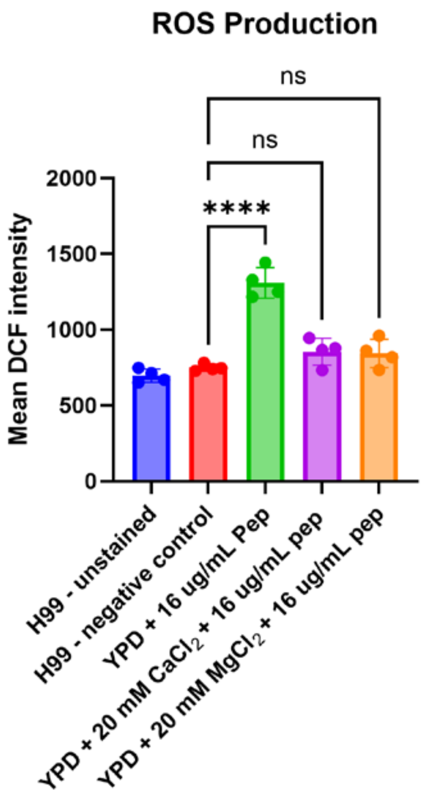

C. albicans phenotype against azoles.

Figure S2. Images of *C. albicans* in 96-well plate at different fluconazole concentrations in RPMI1640 after 48 hr incubation at 37 °C. Image recorded on Nikon eclipse TS2 inverted microscope. Since there was >20% growth in all azole treated wells, a clear MIC value readout could not be determined.

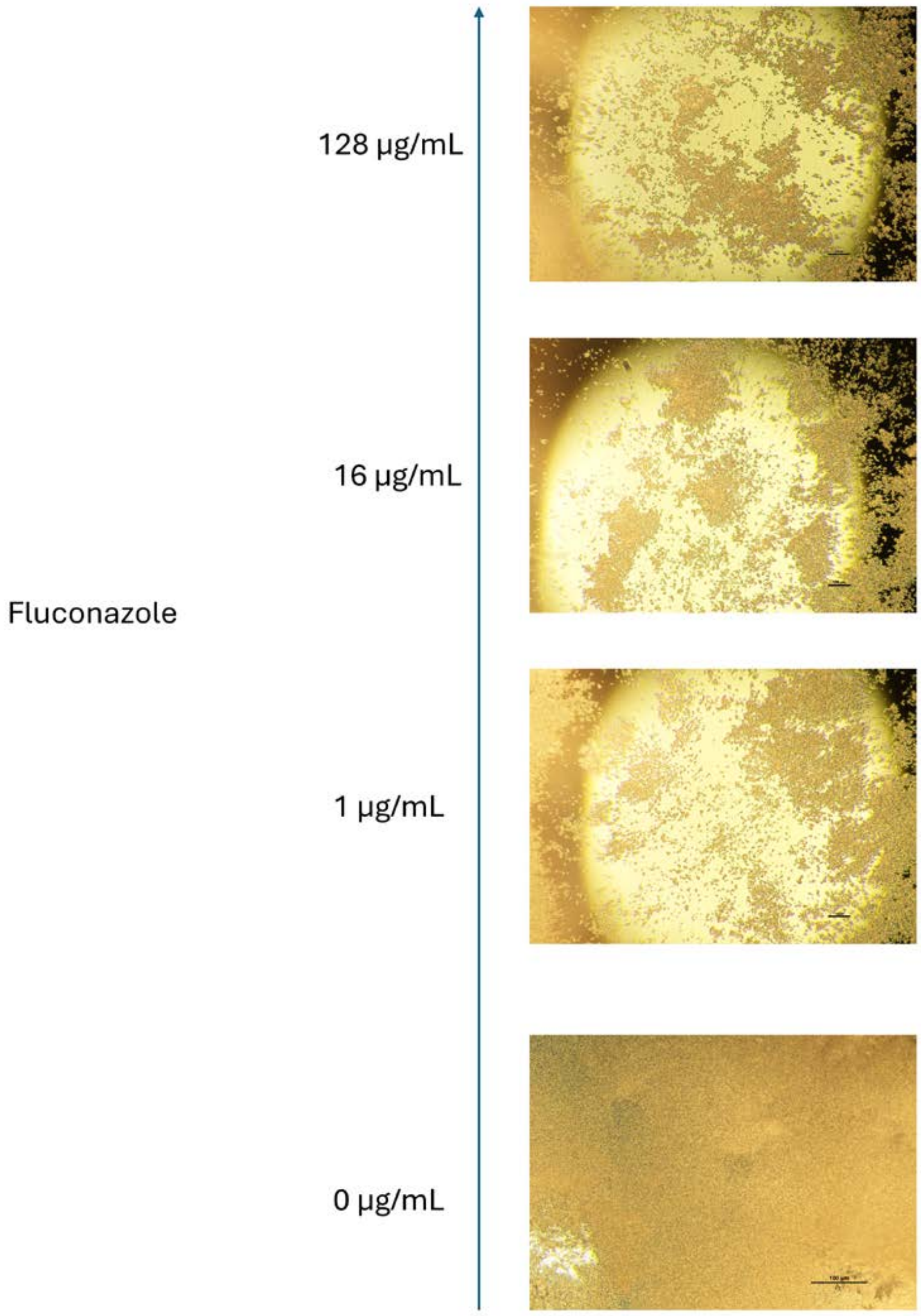

Figure S3. AlphaFold2 model of *Cn* Apt3-Cdc50 complex. Key AFP residues of Cdc50 do not possess the same interactions with Apt3 compared to analogous contact points in the Apt1-Cdc50 complex. Highlighted in the popout, 4.1 Å distance between the side chain functional group of Cdc50 N252 with the side chain functional group of Apt3 T1218.

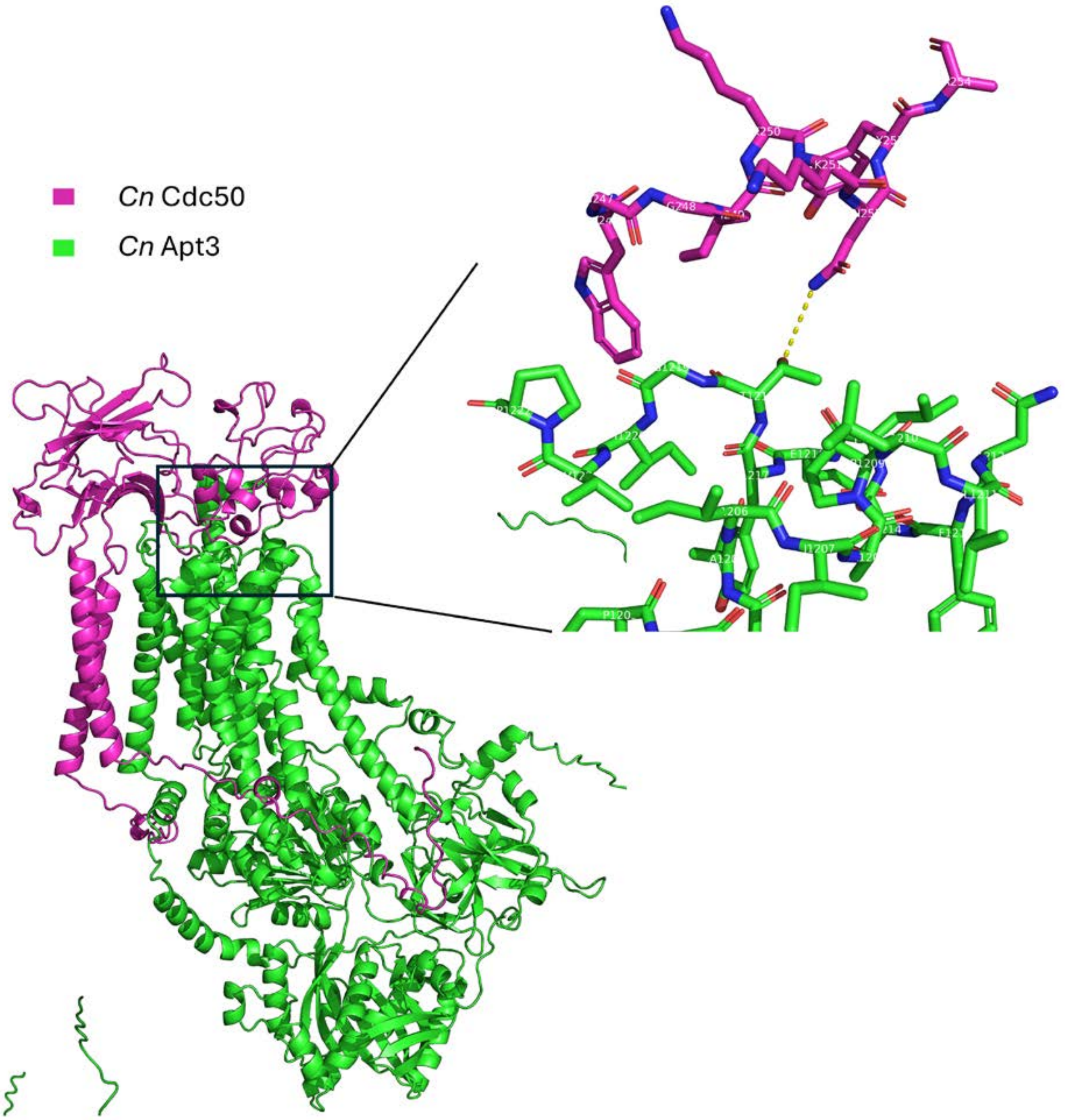

Chemistry

Quality control information provided by GenScript for KKOO-NH2

LCMS

Figure S4. LCMS trace, 220nm, main peak detected at 10.913min, 220 nm = 98.28%.

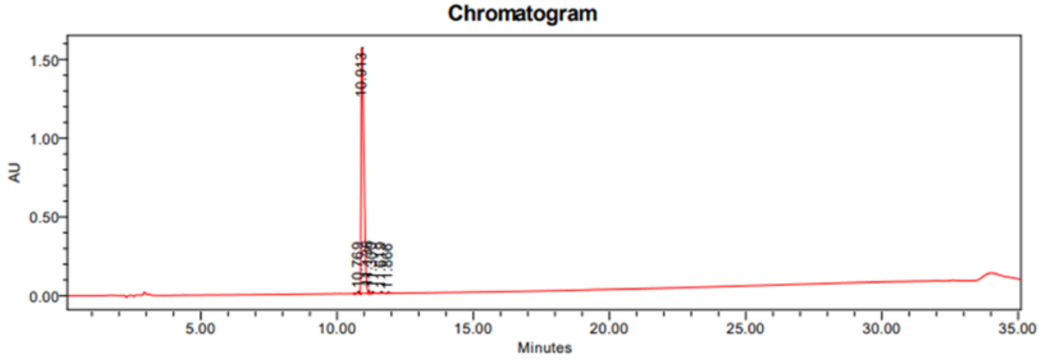

Figure S5. Mass spectrum of main peak for KKOO-NH_2.

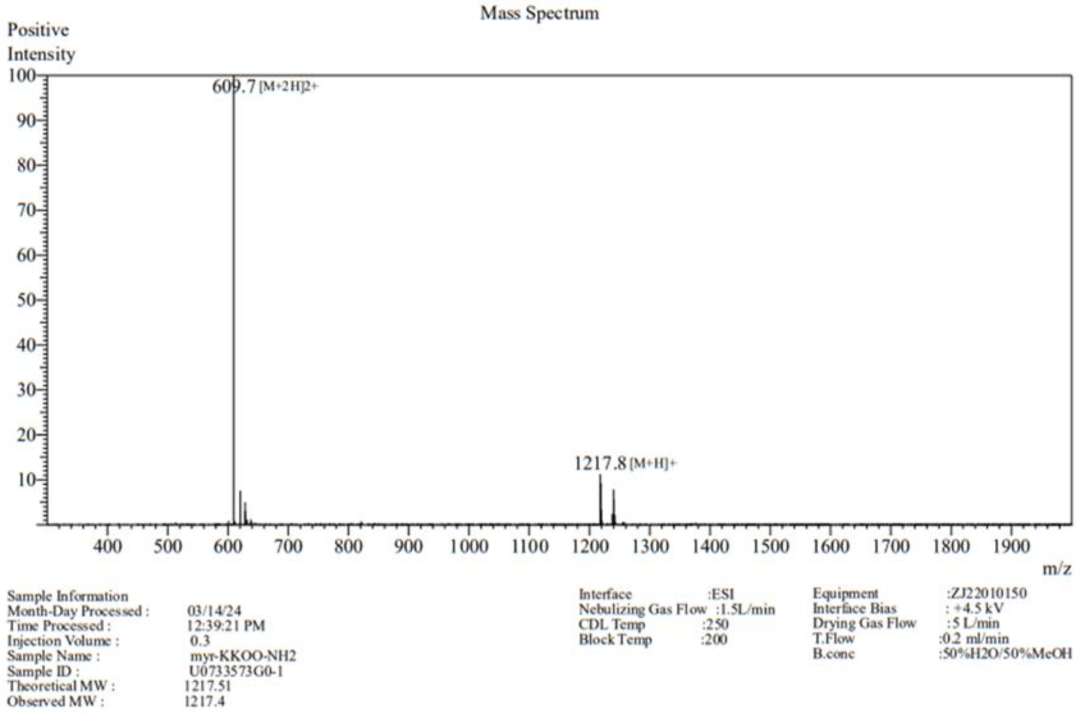

HPLC analysis

Figure S6. HPLC chromatogram of KKOO-NH_2. Detected at 220nm, peptide retention time = 21.918 min, purity = 96.039%, Column = Intersil ODS-3, 4.6*250mm.

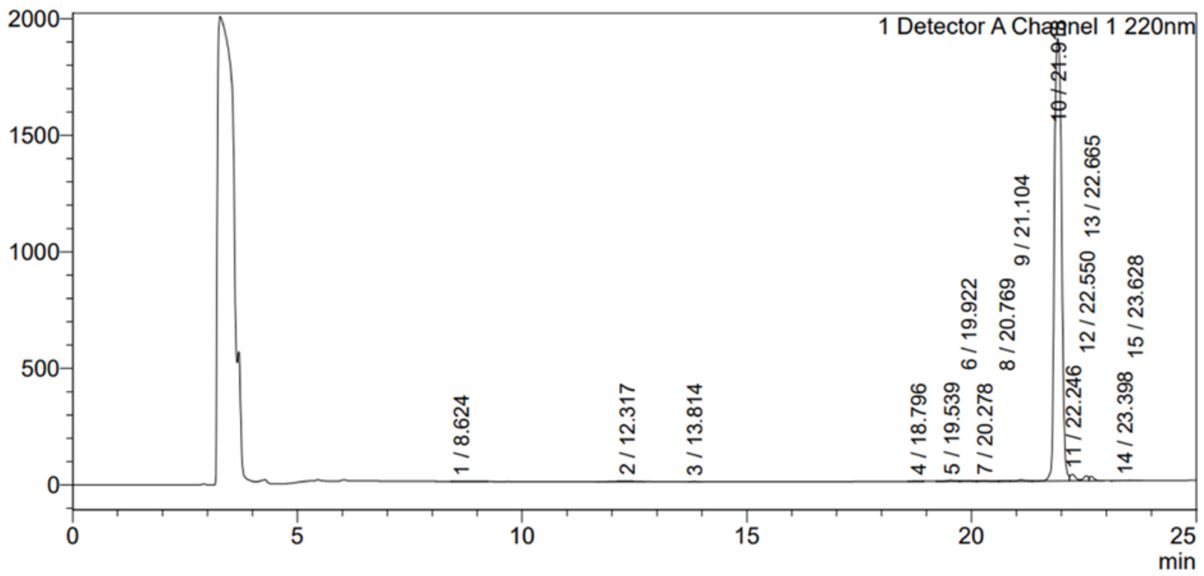

Line A = H2O + 0.065% V/V TFA.

Line B = acetonitrile + 0.05% TFA.

Flow = 1mL/min

Table S10. HPLC program control

Time	Module	Command	Value
0.01	Pumps	B. Conc	5
25.00	Pumps	B. Conc	65
25.01	Pumps	B. Conc	95
27.00	Pumps	B. Conc	95
27.01	Pumps	B. Conc	5
35	Pumps	B. Conc	5
35.01	Controller	stop	

Peptide synthesis of (FITC)KKOO supplemental information

Several versions of the fluorescently labeled peptide were generated, however only the N’ terminal Dap side chain labeled peptide, (Myr-{Dap(FITC)}WIGGOONYA), “(FITC)KKOO” possessed chemical properties amenable for staining yeast cells. The other peptide we attempted to use, (Myr-WIGGOONYAK(FITC)), “KKOO(FITC)” was not soluble in aqueous solution and therefore not reported on further in this study. Upon addition to cells for staining, the C’ terminal lysine side chain labeled version crashed out of solution forming small precipitates. After the FAT was coupled to the N’, Dap side chain was selectively deprotected using 2% hydrazine in DMF for 5 minutes at room temperature on a shaker. In large excess, FITC was conjugated to the side chain of Dap in the presence of DIEA, molar ratios were FITC:DIEA:Peptide, >50:6:1, in DMF. FITC coupling reaction was maintained in the dark at room temperature on a shaker and quenched after 5 minutes reaction time. The resin was rinsed with excess DMF to remove any unreacted FITC prior to washing with methanol and DCM.

Table S11. Solid phase peptide synthesis microwave settings

**Protocol**	**Time** *min:sec*	**Hold** *min:sec*	**Temp** *°C*	**Power** *W*
Coupling	0:00	0:10	50	40
Coupling	0:10	5:00	60	20
Deprotection	0:00	0:10	60	90
Deprotection	0:10	2:00	70	70

Characterization of original ornithine substituted peptides generated in-house

HPLC and LCMS analysis

Table S12. Tabulated material specifications for initial peptides identified.

ID	Retention time *min*	Yield *%*	Purity *(220nm)*	Observed *m/z*	Comment
KKOO	6.652	67.3	>95%	609.9, 1218.6	+2, +1
K6O	6.813	66.0	>95%	616.9, 1232.6	+2, +1
K5O	6.812	67.6	>95%	616.9, 1233.6	+2, +1

Figure S7. UV-Vis absorbance spectrum overlay of the various ornithine substituted peptides. Spectral signals were extracted from the time point of maximum signal intensity of the baseline corrected 3D data matrix recorded on the HPLC-PDA. Characteristic UV-Vis absorbance peaks were observed corresponding to the peptide bonds at 220 nm, and the aromatic side chains at 280 nm. For visualization purposes, trace representing KKOO was offset by a constant of + 0.01 au, K6O was offset by a constant of + 0.3 au and K5O was offset by a constant of + 0.6 au. This figure was prepared with Microsoft Excel.

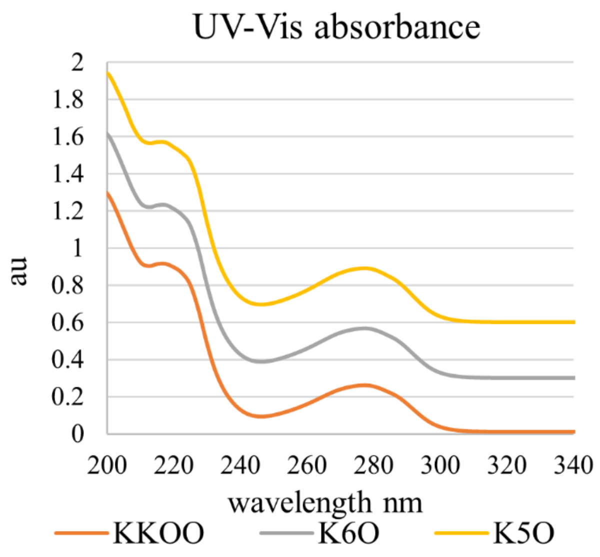

Figure S8. HPLC-PDA and LC-MS of first 3 ornithine substituted peptides. For visualization purposes each HPLC trace was offset by a constant value of + 0.01 au for the 220 nm channel and 0.001 au for the 280 nm channel. Mass spec signal for each sample is directly adjacent to the corresponding sample's HPLC trace. This figure was prepared using Microsoft Excel.

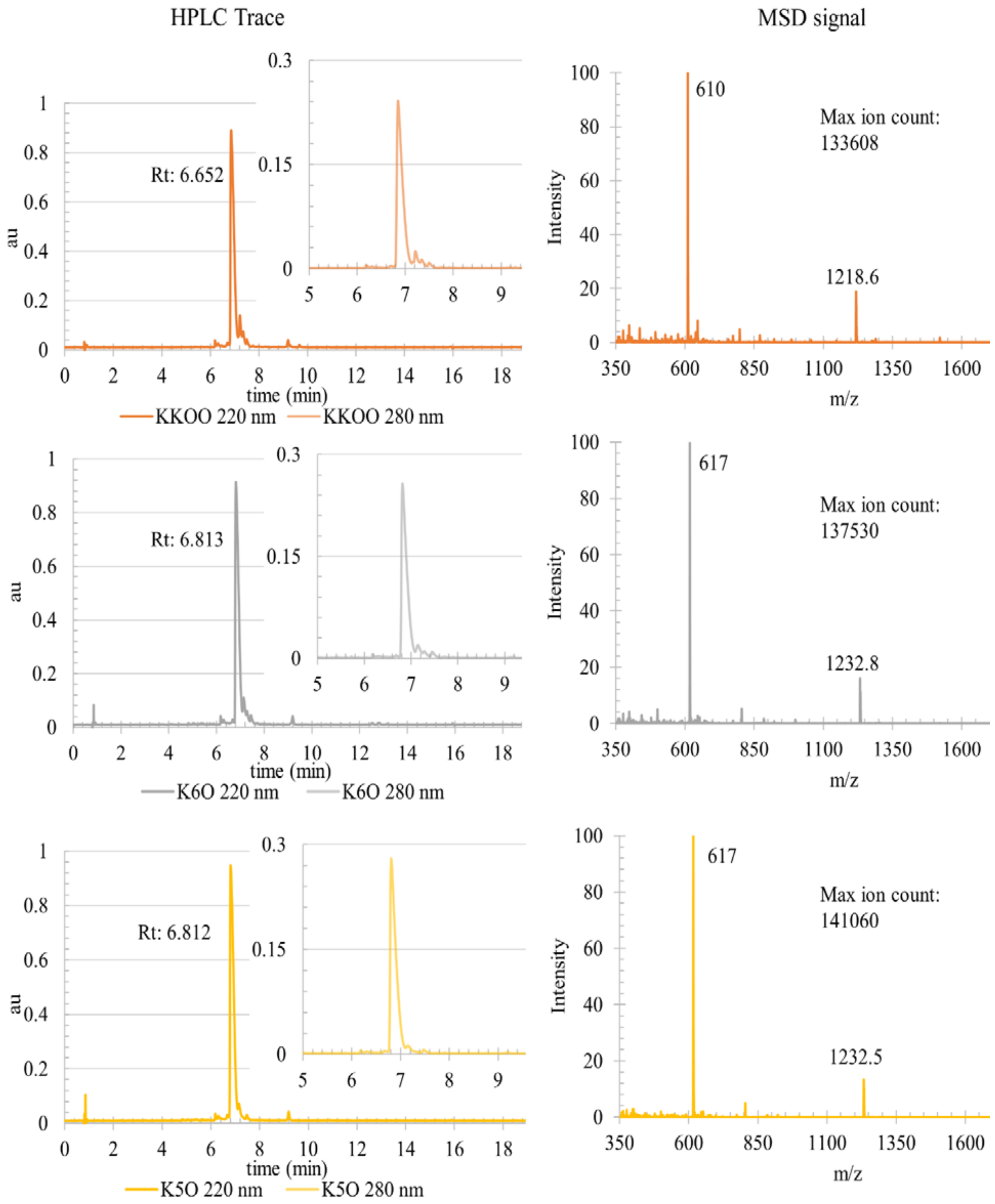

NMR spectroscopy of KKOO

1D and 2D H^1 NMR spectra of KKOO peptide were acquired at sample concentration of 15mg/mL in D_6-DMSO. Data was acquired in a 3mm tube used coaxially in a 5mm probe in a 500 MHz NOVA Varian NMR spectrometer. Sample chamber was maintained at 30 °C for all data acquisition. H^1 and H-H gCOSY spectra were processed and analyzed using MNOVA software. The following post-acquisition processing was performed for single dimension H^1 spectra: A) baseline smoothing (full Whittaker), B) zero filling and linear predict to 64K points C) automatic peak assignment. H^1 aliphatic and aromatic spectral expansions also had apodization applied prior to using the automated coupling assignment tool to analyze the coupling constants more easily. D) The following apodizations were applied to these expansions alone: exponential = -1.2, gaussian = 0.8

Figure S9. Full H^1 spectrum of KKOO. Spectral window acquired from 0 – 11 ppm. Proton spectra acquired with 16 scans, 30° pulse angle, relaxation delay of 2 seconds, no spin, 32K points. Linear predict and zero filling applied to 64K points. No additional apodization or phasing applied.

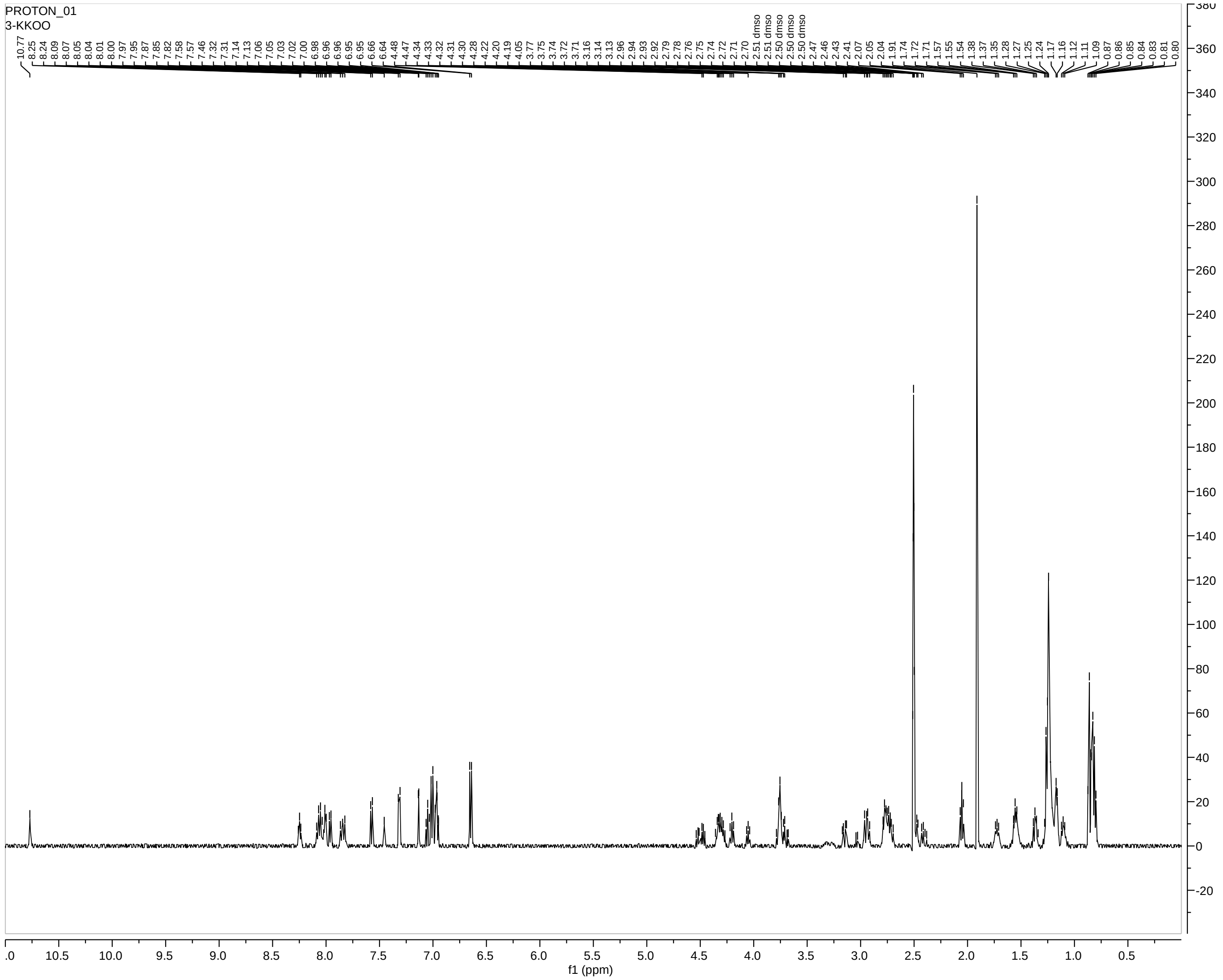

Figure S10. H[1] spectrum of KKOO, aliphatic region expansion. Apodization applied prior to assinging coupling constants using the MNOVA automatic assignment feature.

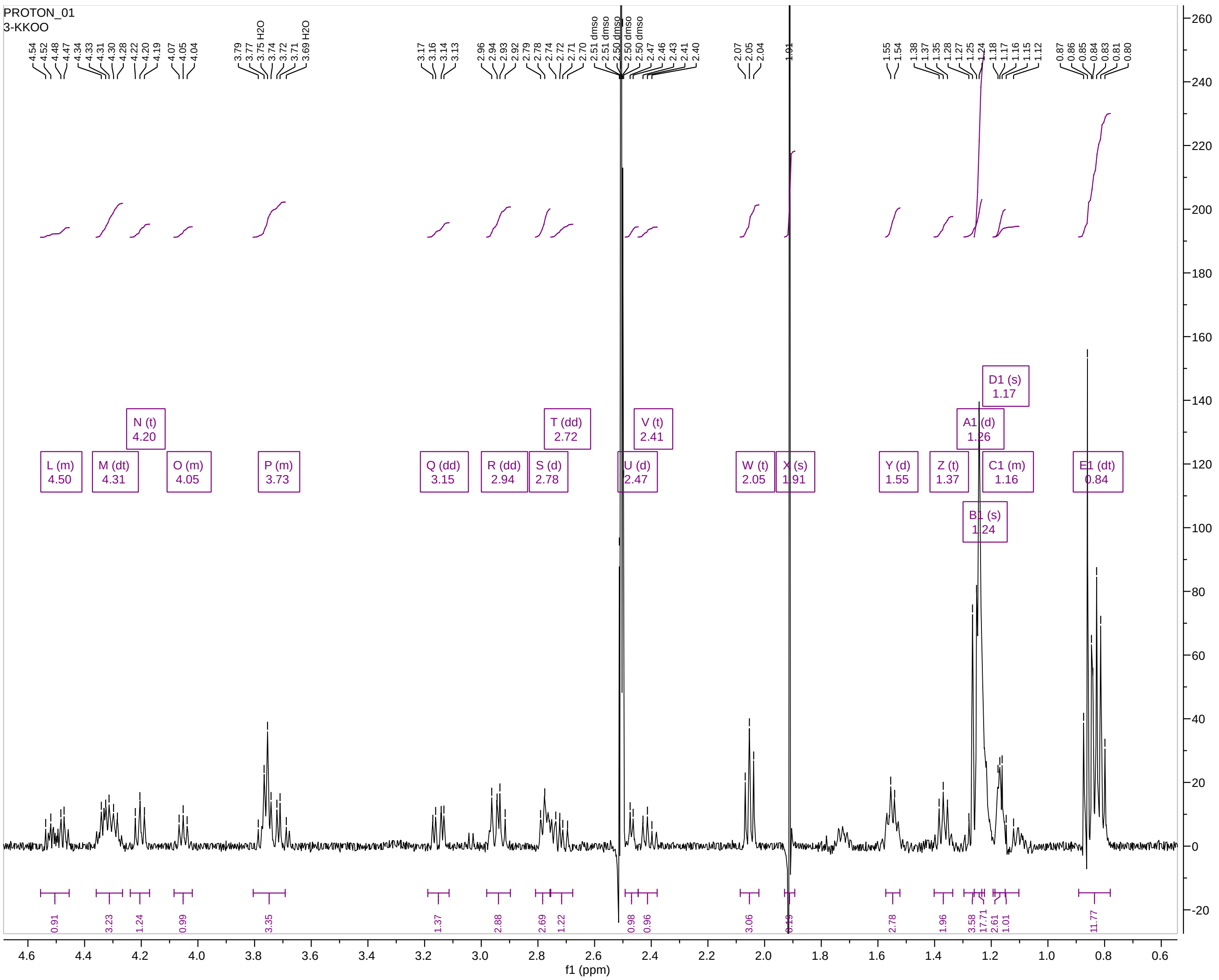

Figure S11. H[1] spectrum, aromatic expansion of KKOO. Apodization applied prior to assinging coupling constants with MNOVA automatic assignment feature.

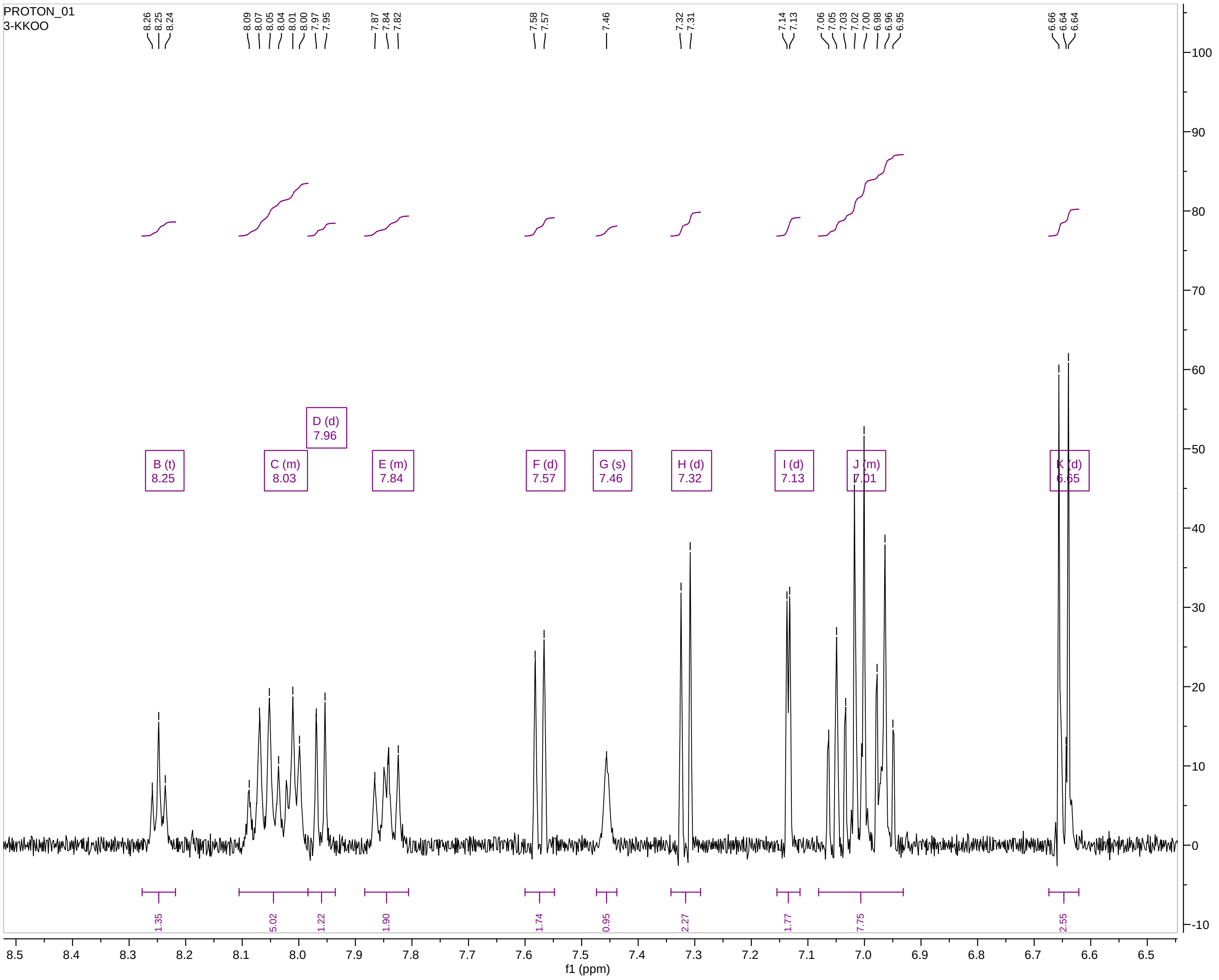

Figure S12. H1 gCOSY of KKOO. This spectra was acquired with spectral window in f2 from 0-9ppm, 512 scans, 1024 increments, 4K complex points. Baseline smoothing applied, no additional apodization applied.

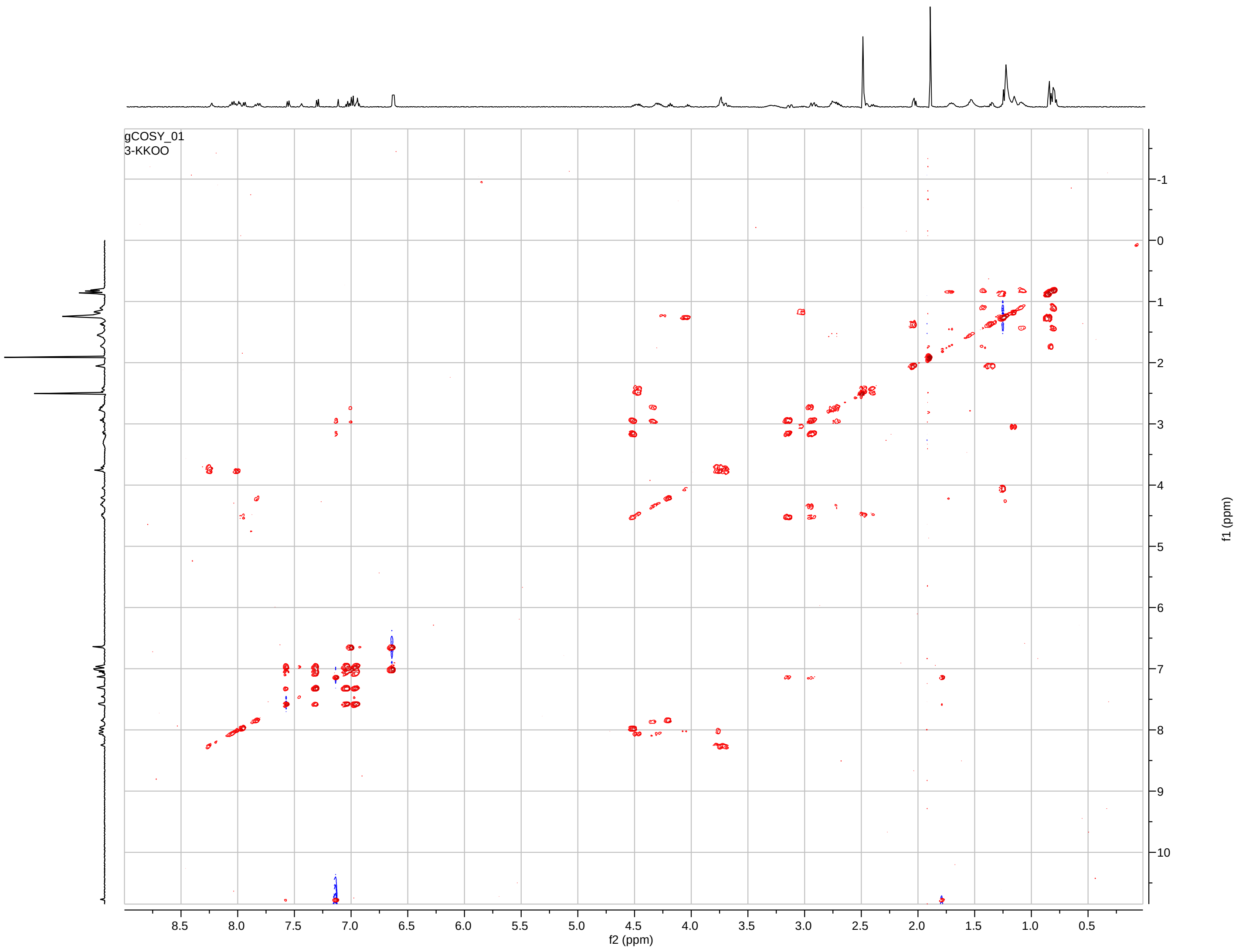

Supplement: Supplementary file 1 — id4c00681_si_001.pdf [file id4c00681_si_001.pdf]
